# Mitochondrial genomes from RNA-Seq reveal phylogeny and selection in *Mepraia* (Hemiptera: Reduviidae)

**DOI:** 10.1007/s00438-026-02434-y

**Published:** 2026-05-19

**Authors:** Matheus Cardoso de Siqueira e Silva, José Paulo Leite Guadanucci, Tiago Belintani

**Affiliations:** 1 Universidade de Araraquara (Uniara), Araraquara, SP Brazil; 2 Rio Claro Arachnology Laboratory, Department of Biodiversity, Institute of Biosciences, São Paulo State University (Unesp), Rio Claro, Brazil

**Keywords:** Mitochondrial genome, Phylogenomics, RNA-seq, Chagas disease vector, Evolutionary adaptation

## Abstract

Despite the epidemiological importance of triatomine bugs as vectors of *Trypanosoma cruzi*, mitogenomic resources remain limited for many lineages, restricting evolutionary and comparative analyses. The endemic Chilean genus *Mepraia* is a notable example, with scarce mitochondrial genomic information despite its relevance to Chagas disease ecology and vector evolution. Here, we reconstructed partial mitochondrial genomes, including all 13 protein-coding genes, from RNA-Seq data for three *Mepraia* species. Phylogenetic analyses confirmed the monophyly of *Mepraia* and resolved interspecific relationships, whereas comparative analyses revealed heterogeneous patterns of intraspecific diversity, with elevated variation in *M. parapatrica*. Codon-based tests detected pervasive purifying selection together with localized signals of positive selection in oxidative phosphorylation genes, particularly NAD5, NAD6, NAD1, and COIII. These results show that transcriptomic data provide a reliable source for mitochondrial genome reconstruction in non-model insects and that adaptive signals can coexist with strong functional constraint in triatomine mitogenomes. Overall, this study expands mitogenomic resources for Triatominae, improves evolutionary inference in *Mepraia*, and provides a foundation for future integrative studies on diversification, mitochondrial evolution, and vector biology.

**Supplementary Information:**

The online version contains supplementary material available at 10.1007/s00438-026-02434-y.

## Introduction

Blood-feeding triatomine bugs are of major biological and public health interest because they include vectors of *Trypanosoma cruzi*l Chagas, 1909 (Kinetoplastida: Trypanosomatidae), the causative agent of Chagas disease. Understanding their evolutionary history is important not only for taxonomy and comparative biology, but also for interpreting patterns of diversification, geographic structure, and vector-associated traits. Among the molecular resources available for such questions, mitochondrial genomes are especially useful because they combine broad phylogenetic utility with sensitivity to population-level and adaptive processes (Eisen and Fraser 2003; Duchêne et al. 2011). However, mitogenomic data remain scarce for several triatomine lineages, including the endemic Chilean genus *Mepraia*, limiting evolutionary inference in a medically relevant group.

High-throughput sequencing (HTS) technologies, such as whole-genome shotgun sequencing (WGS), anchored hybrid enrichment (AHE), transcriptome (RNA-seq), and ultraconserved elements (UCE) capture, have greatly expanded the methods available for recovering mitogenomes (Raposo do Amaral et al. 2015; Moreira et al. 2015; Baeza et al. 2025). RNA-Seq allows for efficient in silico assembly of mitochondrial genomes in non-model organisms, overcoming challenges related to poor DNA quality and complex nuclear genomes (Tian and Smith 2016; Forni et al. 2019; Yang et al. 2024). Despite these advances, most available mitogenomic data are limited to a small number of model or economically significant insect groups, leaving substantial gaps for many other taxa (Cameron 2014; Ramírez et al. 2026). Such taxonomic bias limits comparative and evolutionary analyses across insect lineages. The subfamily Triatominae Jeannel, 1919 consists of blood-feeding insects known for their role as vectors of trypanosomatid parasites, including * T. cruzi* and *Trypanosoma rangeli* Tejera, 1920 (Lent and Wygodzinsky 1979). These insects are predominantly found throughout the Americas, ranging from the southern United States to Central and South America, with some species also present in Asia and Oceania (Monteiro et al. 2018). Triatominae currently includes over 158 species classified into eight tribes and 19 genera (Paiva et al. 2022; Masonick et al. 2025; Oliveira-Correia et al. 2024). Of these, the tribe Triatomini Jeannel, 1909 is the most species-rich and includes several genera of epidemiological significance, such as *Triatoma* Laporte, 1832, *Panstrongylus* Berg, 1879, and *Rhodnius* Stål, 1859.

The genus *Mepraia* Mazza, Gajardo & Jörg, 1940 consists of three species endemics to Chile: *Mepraia spinolai* (Porter, 1933), *M. gajardoi* Frías, Henry & González, 1998, and *M. parapatrica* Frías-Lasserre 2010. These species exhibit distinct ecological traits and geographic distributions and have therefore become the focus of increasing research interest due to their evolutionary and epidemiological relevance (Frías-Lasserre et al. 2018; 2019). *Mepraia* has been investigated using morphological, morphometric, cytogenetic, and molecular approaches, providing strong evidence for the distinctiveness of the three recognized species (Campos-Soto et al. 2011, 2013; Frías-Lasserre 2010; Frías-Lasserre et al. 2019). Although the monophyly of *Mepraia* is well supported (Kieran et al., 2021; Campos-Soto et al., 2022), recent phylogenomic evidence points to rapid diversification and historical introgression among lineages, suggesting that ancient hybridization played a role in shaping present-day genetic structure (Belintani et al. 2023).

Beyond their evolutionary relevance, *Mepraia* species are epidemiologically important in Chile as components of the sylvatic transmission cycle of Chagas disease and represent the only endemic triatomine genus involved in *T. cruzi* circulation in wild habitats (Tapia-Garay et al. 2018; Lambarri et al. 2018). *Mepraia spinolai* is the most widespread and frequently infected species, with high natural infection rates and documented feeding on mammals and humans, highlighting its major epidemiological role (Coronado et al. 2009; Espinoza et al. 2013). *Mepraia gajardoi* has also been reported as naturally infected in northern coastal and peridomestic environments, indicating potential involvement in local transmission dynamics (González et al. 2015; Tapia-Garay et al. 2018). In contrast, *M. parapatrica* occupies more restricted coastal and insular habitats; although less frequently documented, infected individuals recorded on offshore islands confirm its participation in the sylvatic cycle of *T. cruzi* (Rives-Blanchard et al. 2017).

Despite the medical importance of Triatominae and the increasing availability of genomic data for related taxa, mitochondrial genomic information remains limited (Ramírez et al. 2026). At the same time, large amounts of publicly available transcriptomic data offer an underexplored opportunity to recover mitochondrial sequences without the need for additional sequencing efforts. In this study, we reconstruct partial mitochondrial genomes of *M. gajardoi*, *M. parapatrica*, and *M. spinolai* using RNA-Seq datasets retrieved from public repositories. By integrating these newly recovered mitochondrial sequences into comparative phylogenetic and evolutionary analyses, we aim to clarify interspecific relationships and help fill important gaps in the evolutionary understanding of *Mepraia*. The genomic resources generated here demonstrate the potential of RNA-Seq data as a valuable source for mitogenome reconstruction and provide a foundation for future studies of mitochondrial diversity, lineage diversification, and evolutionary dynamics in this medically relevant genus.

## Materials and methods

### Taxon sampling and mitogenome assembly from RNA-Seq data

RNA-Seq data used in this study were previously generated and published by Belintani et al. (2023) and are publicly available in the National Center for Biotechnology Information (NCBI) Sequence Read Archive (SRA) under BioProject accession PRJNA916468. Briefly, head and salivary gland tissues were carefully dissected from fifth-instar male nymphs of *Mepraia gajardoi* (n = 6), *M. spinolai* (n = 6), and *M. parapatrica* (n = 6), ensuring preservation of salivary gland integrity (Table S1). Specimens were collected from natural populations within the known geographic range of each species, representing their typical ecological contexts, as detailed in Belintani et al. (2023).

In that study, total RNA was extracted individually from each specimen using a Trizol/chloroform protocol (Chomczynski and Mackey 1995). RNA quality was initially assessed by agarose gel electrophoresis and quantified using a Qubit fluorometer, while NanoDrop spectrophotometry was used to select samples with 260/230 and 260/280 absorbance ratios close to 2.0. RNA integrity was further evaluated by capillary electrophoresis using an Agilent 2100 Bioanalyzer, yielding RNA integrity numbers (RIN) of 7.1 ± 0.3 across the 18 samples. Libraries were then prepared using the TruSeq® RNA Sample Prep Kit v2 (Illumina) and sequenced on an Illumina HiSeq 2500 platform with 2 × 100 bp paired-end reads at the Functional Genomics Laboratory of ESALQ–USP (Brazil). No additional RNA extraction or sequencing was performed for the present study.

In the present study, raw sequencing reads were quality-assessed using FastQC v0.11.9 (Andrews 2010) and trimmed with Trimmomatic v0.39 (Bolger et al., 2014) using the following parameters: LEADING:5, TRAILING:5, SLIDINGWINDOW:5:20, and MINLEN:50.

Partial mitochondrial genomes were reconstructed using an iterative baiting and mapping strategy implemented in MITObim v1.9.1 (Hahn et al. 2013). The mitochondrial genome of *Triatoma infestans* (NC_035547) was used as the initial reference due to its close phylogenetic relationship to *Mepraia,* as well as its completeness and high-quality annotation. During the iterative assembly process, the reference sequence is progressively updated, reducing potential bias from the initial reference.

Paired-end reads were merged with FLASH (Magoč and Salzberg 2011) to recover extended fragments when possible. Assemblies were generated using the integrated MIRA assembler v3.4.1.1, with a maximum of 30 iterative cycles; in most cases, convergence was achieved within five iterations. For protein-coding genes that were fragmented or poorly recovered, MitoGeneExtractor v1.9.5 (Brasseur et al. 2023) was additionally employed to improve the recovery of mitochondrial protein-coding genes (PCGs). Final contigs were annotated using MITOS (Bernt et al., 2013) under the invertebrate mitochondrial genetic code. The resulting mitochondrial maps represent partial reconstructions derived solely from expressed PCGs. As a result, mitochondrial genome structure and gene order were not inferred, and structural validation by PCR was not conducted.

All computational analyses were performed using high-performance computing resources provided by the Center for Scientific Computing (NCC/GridUNESP) at São Paulo State University.

### Phylogenetic analyses

Phylogenetic analyses were conducted to investigate evolutionary relationships within the genus *Mepraia* and to determine its placement within the subfamily Triatominae using 13 mitochondrial protein-coding genes (PCGs). Two datasets were assembled: (i) a focal dataset including PCGs recovered in this study from all individuals of *M. gajardoi*, *M. parapatrica*, and *M. spinolai*; and (ii) an expanded dataset that combined the focal dataset with 51 additional Triatominae mitogenomes retrieved from GenBank (Table S2).

Two analytical strategies were applied. First, a concatenated analysis of the 13 PCGs from the focal dataset was conducted to resolve intrageneric relationships within *Mepraia*. Second, the expanded dataset was used to infer the phylogenetic placement of *Mepraia* within a broader Triatominae context.

Multiple sequence alignments for each PCG were generated using MAFFT v7.475 (Katoh and Standley 2013) under the E-INS-i strategy. This approach applies global pairwise alignment with iterative refinement and is suitable for datasets containing conserved and variable regions with indels. Alignments were manually inspected in MEGA11 (Tamura et al. 2021) to confirm reading-frame integrity, detect internal stop codons, and verify codon consistency. Ambiguously aligned positions were filtered using TrimAl v1.2 (Capella-Gutiérrez et al. 2009) with a gap threshold of 0.8 (-gt 0.8), removing alignment columns containing more than 80% gaps. The mitochondrial PCG alignments were concatenated into a partitioned supermatrix using AMAS v1.0 (Borowiec 2016). Gene boundaries were retained during concatenation, and a partition file specifying the exact nucleotide coordinates of each locus was generated simultaneously.

Phylogenetic inference under the maximum likelihood (ML) criterion was conducted using IQ-TREE 3 (Wong et al. 2025) on the concatenated mitochondrial dataset. The best-fitting substitution model and partitioning scheme were jointly estimated using ModelFinder Plus (-m MFP + MERGE; Kalyaanamoorthy et al. 2017), allowing automatic merging of partitions with similar evolutionary patterns under a relaxed clustering algorithm (–rcluster 10). Branch support was evaluated using 1000 replicates of the SH-like approximate likelihood ratio test (SH-aLRT; Guindon et al. 2010) and 1000 ultrafast bootstrap replicates (UFBoot; Hoang et al. 2018). Numerical stability was ensured using the safe likelihood optimization mode (–safe), and ultrafast bootstrap branch length optimization was refined using the BNNI correction (–bnni) to reduce potential support overestimation.

Bayesian inference (BI) was conducted in MrBayes v3.2.7 (Ronquist et al. 2012) on the same concatenated dataset. The ML partitioning scheme was retained for Bayesian inference, with all partitions analyzed under GTR + I + Γ (nst = 6; rates = invgamma). Model parameters were unlinked across partitions, and rate heterogeneity among partitions was accommodated using ratepr = variable. Two independent runs were performed, each with four Markov chains, for 20 million generations, sampling every 1,000 generations. Convergence was assessed by monitoring the average standard deviation of split frequencies (ASDSF < 0.01) and by examining effective sample sizes (ESS > 200) and parameter trace plots in Tracer v1.7.2 (Rambaut et al. 2018). Log-likelihood traces indicated that stationarity was reached after approximately 5 million generations. A conservative burn-in of 25% was applied to exclude pre-convergence samples. Posterior probabilities (PP) were calculated from the remaining trees to estimate nodal support.

Topological congruence between maximum likelihood (ML) and Bayesian inference (BI) trees was assessed with particular emphasis on relationships within *Mepraia* and among major Triatominae lineages. Nodes were considered strongly supported when they received both ultrafast bootstrap values (UFBoot) ≥ 95% and posterior probabilities (PP) ≥ 0.95. Final trees were visualized and annotated in FigTree v1.4.4 (Rambaut 2012).

### Genetic divergence and selection analyses of mitochondrial protein-coding genes

Pairwise genetic distances were estimated for each of the 13 mtDNA PCGs of *Mepraia* using the Kimura 2-Parameter (K2P) model (Kimura 1980) with a gamma distribution (shape parameter = 1) to account for rate heterogeneity among sites. The K2P model was selected because it is widely adopted in Triatominae studies and provides an appropriate balance between simplicity and accuracy for mitochondrial (Monteiro et al. 2013; Belintani et al. 2020; Silva et al. 2023). Nucleotide sequences (all codon positions) were aligned prior to distance estimation, and standard errors were calculated via 1000 bootstrap replicates. Analyses were performed in MEGA11 (Tamura et al. 2021).

Genetic structure was explored through a principal component analysis (PCA) based on the K2P distance matrix. PCA was conducted in PAST v4.03 (Hammer et al. 2001) to visualize patterns of variation among individuals in multivariate space and to assist in assessing intra- and interspecific relationships.

Selection patterns and variation in evolutionary rates across the 13 mtDNA PCGs were assessed using codon-based models implemented in HyPhy v2.5 (Kosakovsky Pond et al. 2005, 2020). Three complementary approaches were applied to detect both site-specific and lineage-specific selection.

The Mixed Effects Model of Evolution (MEME) was used to identify sites subject to episodic diversifying selection across the phylogeny (Murrell et al. 2012), employing four ω rate classes and a significance threshold of *p* < 0.05 The Fast Unconstrained Bayesian Approximation (FUBAR) method was then used to detect sites under pervasive positive or purifying selection (Murrell et al. 2013), using 40 grids points and 2 million MCMC iterations, with the first 20% discarded as burn-in; two independent runs were conducted to verify convergence. Finally, the Branch-site Unrestricted Statistical Test for Episodic Diversification (BUSTED) was applied to detect branches experiencing episodic positive selection (Murrell et al. 2015). The model incorporated synonymous rate variation (SRV) with four classes plus an additional class to account for alignment noise, and significance was assessed using a likelihood ratio test (*p* < 0.05).

### Data availability

The mitochondrial sequences generated in this study are publicly available in GenBank (NCBI) under accession numbers PV521367–PV521600 (see Table S4). Additional data and materials related to this study are available from the authors upon reasonable request.

## Results

**Fig. 1:**
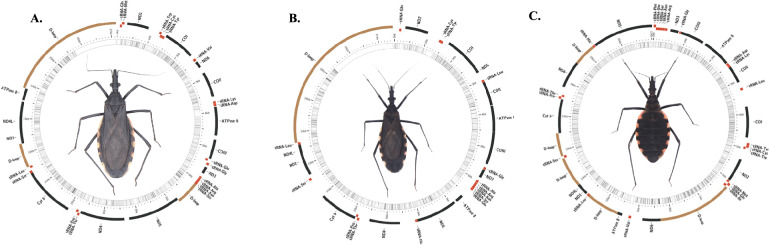
Partial mitochondrial genome maps reconstructed from RNA-Seq data for the three species of *Mepraia*. **A** *M. gajardoi*, **B** *M. parapatrica*, and **C** *M. spinolai*. Circular diagrams show the arrangement and orientation of mitochondrial genes recovered in the assemblies, including the 13 protein-coding genes (PCGs), transfer RNAs (tRNAs), and ribosomal RNAs (rRNAs) when present. Genes on the majority and minority strands are shown on the outer and inner tracks, respectively. Black segments indicate protein-coding genes, red segments indicate tRNAs, and brown segments indicate rRNAs. These maps represent partial mitochondrial assemblies; therefore, tRNA and rRNA genes are not fully recovered, and gene order does not necessarily reflect the complete mitochondrial genome

The mitochondrial genomes of *M. gajardoi*, *M. parapatrica*, and *M. spinolai* were assembled from previously generated RNA-Seq datasets using an iterative baiting and mapping strategy, which reduces potential bias associated with the initial reference sequence. A total of 18 partial mitochondrial assemblies were recovered (six per species) using a reference-guided strategy. Although the assemblies are partial due to gaps in transcript coverage, all 13 mitochondrial protein-coding genes (PCGs) were successfully reconstructed in every sample. The three highest-quality assemblies are shown in Fig. 1A–C.

The assembled mitochondrial sequences ranged in length from approximately 15,173 bp to 17,762 bp (Table 1), values consistent with mitochondrial genome sizes reported for Triatominae (Pita et al. 2017; Cigarroa-Toledo et al. 2024; Almeida et al. 2025). As expected from transcriptome-based assemblies, recovery of non-protein-coding regions was variable. While all 13 PCGs were consistently recovered, the detection of tRNA and rRNA genes varied among assemblies and is most likely attributable to uneven transcript representation rather than true biological differences.

**Table 1:** Descriptive statistics of mitochondrial assemblies for *Mepraia gajardoi*, *M. parapatrica*, and *M. spinolai*

Species	Samples	Length (bp)	Reads (n)	Max coverage ( ×)	Mean coverage ( ×)	GC content (%)	T content (%)
*M. spinolai*	*M. spinolai1*	17,529	1,564,229	298,069	11,000	29.6	66.2
*M. spinolai*	*M. spinolai2*	15,975	1,096,992	452,552	9,157	29.9	64.1
*M. spinolai*	*M. spinolai3*	16,941	1,716,780	577,372	13,562	30.3	62.5
*M. spinolai*	*M. spinolai4*	17,195	1,721,226	450,774	13,887	29.9	62.1
*M. spinolai*	*M. spinolai5*	17,198	1,628,346	340,765	11,461	29.0	62.1
*M. spinolai*	*M. spinolai6*	17,165	1,621,226	350,774	12,886	29.7	62.0
*M. gajardoi*	*M. gajardoi7*	17,762	858,105	242,684	5,925	29.3	62.1
*M. gajardoi*	*M. gajardoi8*	15,898	940,275	301,135	7,237	29.3	66.8
*M. gajardoi*	*M. gajardoi9*	16,398	1,146,516	264,059	9,346	29.2	62.5
*M. gajardoi*	*M. gajardoi10*	17,173	1,099,046	270,813	7,744	29.3	63.0
*M. gajardoi*	*M. gajardoi11*	17,398	1,244,312	264,054	8,246	29.2	62.5
*M. parapatrica*	*M. gajardoi12*	15,173	1,198,077	260,813	7,744	29.3	63.0
*M. parapatrica*	*M. parapatrica13*	16,492	1,201,222	312,731	7,500	29.3	59.9
*M. parapatrica*	*M. parapatrica14*	16,813	811,235	252,299	6,224	29.1	60.7
*M. parapatrica*	*M. parapatrica15*	17,492	901,222	328,73	6,500	29.3	58.9
*M. parapatrica*	*M. parapatrica16*	15,813	711,225	202,299	5,724	29.7	61.9
*M. parapatrica*	*M. parapatrica17*	16,27	1,240,510	378,017	10,097	29.2	57.4
*M. parapatrica*	*M. parapatrica18*	17,094	742,086	257,044	5,470	29.0	57.4

Assembly length corresponds to the total length of recovered mitochondrial contigs (bp). Coverage values ( ×) represent sequencing depth per nucleotide position, estimated from mapped RNA-Seq reads. High coverage values reflect the elevated expression of mitochondrial transcripts in RNA-Seq datasets. GC and AT contents were calculated from consensus sequences; due to the presence of undetermined bases (Ns), their sum may not equal 100%

In *M. gajardoi* (17,138–17,762 bp), five tRNA loci were absent or incomplete in the best-quality assembly (trnE, trnH, trnI, trnL2, and trnV). In *M. parapatrica* (15,813–17,492 bp), eight tRNA loci were missing or incomplete (trnD, trnF, trnH, trnI, trnL1, trnS2, trnT, and trnV). Assemblies of *M. spinolai* (15,975–17,529 bp) showed comparatively higher tRNA recovery, with some samples presenting all 22 tRNAs; however, several loci (*trnF*, *trnP*, *trnQ*, *trnT*, and *trnV*) were absent or incomplete in other assemblies. The order and strand orientation of the 13 PCGs were conserved across all samples and were identical to the reference genome, indicating consistent recovery of these genes.

Mitochondrial AT content ranged from 57.41% to 66.76% (Table 1), consistent with the AT-rich composition typical of hemipteran mitogenomes (Wang et al. 2015). Sequencing depth across recovered mitochondrial regions was high, with average coverage values ranging from 5,470 × to over 13,800 × . This high coverage supports the robustness of the reconstructed PCGs for downstream phylogenetic and molecular evolutionary analyses.

### Phylogenetic analyses

**Fig. 2:**
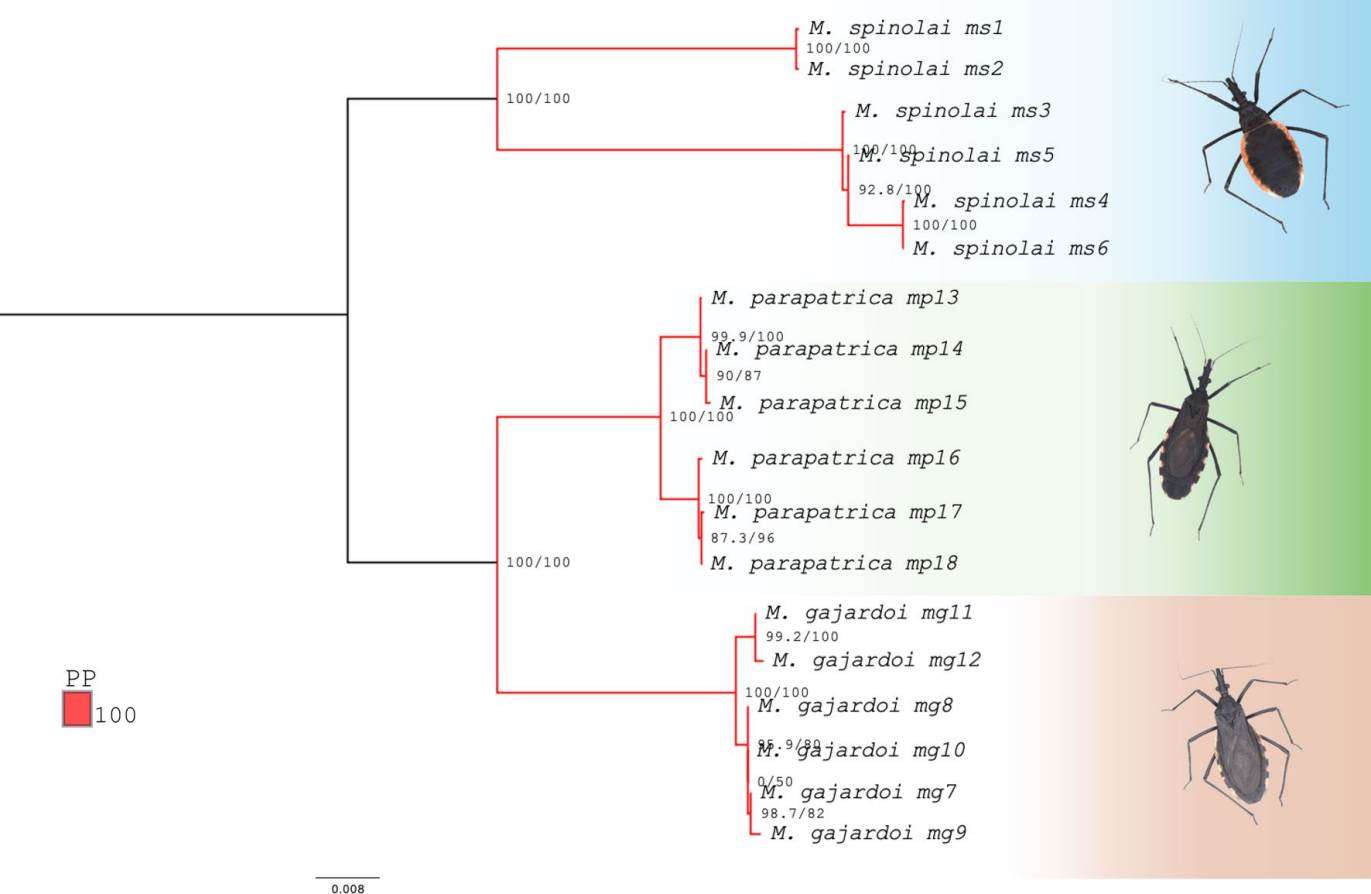
Phylogenetic relationships among individuals of the three *Mepraia* species inferred from a concatenated dataset of the 13 mitochondrial protein-coding genes. The topology was reconstructed using maximum likelihood and Bayesian inference approaches. Node labels indicate branch support values (SH-aLRT / ultrafast bootstrap), and posterior probabilities are indicated by branch coloration. Photographs of representative specimens are shown to the right of each clade, and colored backgrounds highlight species-level groupings

**Fig. 3:**
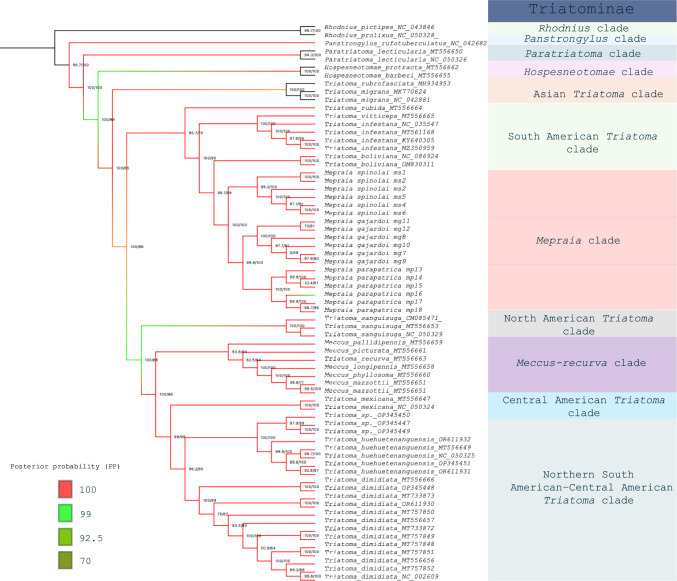
Maximum likelihood phylogeny of Triatominae inferred from a concatenated alignment of the 13 mitochondrial protein-coding genes, including the *Mepraia* sequences generated in this study and additional mitogenomes retrieved from GenBank. Branch support values correspond to SH-aLRT and ultrafast bootstrap replicates, while posterior probabilities from Bayesian inference are represented by branch coloration

Phylogenetic analyses based on the concatenated dataset of 13 mitochondrial PCGs yielded well-resolved topologies with high statistical support for most internal nodes (see Supplementary Fig_S1_Phylogenies_ML_BI_Mepraia.pdf)*.* Both focal (Fig. 2) and comprehensive datasets (Fig. 3) showed strong topological congruence between ML and BI.

In the focal dataset, *Mepraia* exhibited clear and well-supported species-level differentiation (SH-aLRT = 100%, UFBoot = 100%, PP = 1.0, Fig. 2). *Mepraia spinolai*, *M. parapatrica*, and *M. gajardoi* were consistently recovered as distinct genetic clusters, each supported by maximal node support values, indicating strong resolution of species boundaries.

*Mepraia spinolai* exhibited uniformly high internal support, with most nodes receiving maximal values (SH-aLRT = 100%, UFBoot = 100%, PP = 1.0). A single internal node showed slightly reduced ML support (SH-aLRT = 92.8%; UFBoot = 100%), while posterior probability remained maximal (PP = 1.0). *Mepraia parapatrica* was also strongly supported as monophyletic (SH-aLRT = 100%; UFBoot = 100%; PP = 1.0). One internal relationship showed moderate ML support (SH-aLRT = 87.3%; UFBoot = 96%), whereas PP remained high (PP = 1.0). *Mepraia gajardoi* was recovered as a distinct clade with maximal support at the species level. The interspecific relationships were fully resolved, with *M. spinolai* recovered as sister to the clade formed by *M. parapatrica* + *M. gajardoi*, supported by SH-aLRT = 100%, UFBoot = 100%, and PP = 1.0.

In the comprehensive analysis including 69 taxa (Fig. 3), both ML and BI recovered a well-structured phylogeny for Triatominae. *Mepraia* remained strongly supported as a monophyletic lineage (SH-aLRT = 99.9%; UFBoot = 100%; PP = 1.0) and maintained the same internal relationships observed in the focal dataset. The genus was consistently recovered as the sister group to the South American *Triatoma* clade, including species such as *T. infestans*, *T. boliviana*, *T. vitticeps*, and *T. rubida*, within the broader South American radiation of Triatomini. This clade was, in turn, inferred as sister to the major lineage of Central and North American species (SH-aLRT = 100%; UFBoot = 98%; PP = 1.0).

*Rhodnius*, *Panstrongylus*, *Paratriatoma*, and *Hospesneotomae* were recovered as monophyletic with maximal support. Within *Triatoma* sensu lato, the phylogeny revealed a clear geographic structuring, with Asian, South American, North American, Central American, and Northern South American–Central American clades consistently delimited and strongly supported.

Although most deeper relationships were strongly supported, some basal nodes exhibited moderate ML support (SH-aLRT ≈ 70–80%; UFBoot ≈ 70–80%), whereas posterior probabilities remained high (PP ≥ 0.95) (see Supplementary Fig_S1_Phylogenies_ML_BI_Mepraia.pdf)*.* No strongly supported clades (UFBoot ≥ 95% and PP ≥ 0.95) showed topological conflict between ML and BI, and minor incongruences were restricted to deeper nodes with reduced ML support.

### Genetic distances, evolutionary rate, and selection analyses

Principal component analyses (PCA) revealed consistent patterns of nucleotide variation across mitochondrial protein-coding genes (see Supplementary_PCA.pdf). Although the magnitude of variation differed among loci, the overall multivariate structure was highly concordant across genes and consistently reflected species-level differentiation.

Across all loci, samples were partitioned into three well-defined groups corresponding to *M. gajardoi*, *M. parapatrica*, and *M. spinolai* (Supplementary_PCA.pdf, Figures S1–S13). For most genes (COI, COII, COIII, CytB, NAD1, NAD2, NAD4, NAD4L, NAD5, and NAD6), species separation occurred primarily along the first principal component (PC1), with minimal overlap among clusters. These loci exhibited compact intraspecific groupings and clear interspecific differentiation (Table 2).

**Table 2:** Summary of pairwise Kimura 2-parameter (K2P) genetic distances (substitutions per site) among mitochondrial protein-coding genes in *Mepraia*

Gene	Length (bp)	*M. spinolai*	*M. gajardoi*	*M. parapatrica*	*spinolai–gajardoi*	*spinolai–parapatrica*	*gajardoi–parapatrica*
COI	1,542	0.000–0.000	0.000–0.010	0.000–0.080	0.050–0.060	0.060–0.080	0.060–0.080
COII	687	0.000–0.000	0.000–0.000	0.000–0.090	0.050–0.060	0.080–0.120	0.080–0.100
COIII	784	0.000–0.010	0.000–0.010	0.000–0.090	0.040–0.050	0.090–0.090	0.090–0.090
CytB	1,14	0.000–0.000	0.000–0.020	0.000–0.080	0.050–0.060	0.080–0.090	0.080–0.090
ATP6	684	0.000–0.010	0.000–0.010	0.000–0.080	0.060–0.060	0.080–0.110	0.080–0.100
ATP8	165	0.000–0.010	0.000–0.040	0.000–0.110	0.000–0.160	0.060–0.160	0.040–0.160
NAD1	918	0.000–0.000	0.000–0.010	0.000–0.060	0.050–0.080	0.060–0.080	0.070–0.080
NAD2	1,029	0.000–0.000	0.000–0.010	0.000–0.060	0.050–0.080	0.060–0.080	0.070–0.080
NAD3	357	0.000–0.010	0.000–0.020	0.000–0.130	0.060–0.070	0.070–0.130	0.070–0.100
NAD4	1,379	0.000–0.010	0.000–0.010	0.000–0.080	0.040–0.050	0.080–0.090	0.080–0.090
NAD4L	297	0.000–0.010	0.000–0.010	0.000–0.080	0.040–0.050	0.080–0.090	0.080–0.090
NAD5	1,716	0.000–0.010	0.000–0.010	0.000–0.080	0.050–0.060	0.070–0.080	0.070–0.080
NAD6	531	0.000–0.007	0.000–0.011	0.000–0.045	0.032–0.037	0.039–0.053	0.030–0.045

Intraspecific distances are reported separately for each species, and interspecific distances are shown for all species pairs. Values represent minimum–maximum ranges across individuals

*Mepraia spinolai* consistently formed the most compact cluster across loci, reflecting low intraspecific genetic distances (generally 0.000–0.010 substitutions per site; Supplementary_Distance_genetics.xlsx, Table S1). Slightly higher values were observed in faster-evolving loci such as ATP8, where intraspecific distances ranged from 0.000 to 0.010.

*Mepraia gajardoi* showed moderately broader dispersion in PCA space for some loci, with intraspecific distances reaching 0.000–0.040 in ATP8 and 0.000–0.020 in NAD3, but remaining 0.000–0.010 for most genes.

*Mepraia parapatrica* exhibited comparatively greater intraspecific dispersion for specific loci, particularly ATP8 and NAD3. In ATP8, intraspecific distances ranged from 0.000 to 0.110, whereas in NAD3 values ranged from 0.000 to 0.130, indicating higher variability relative to more conserved loci such as COI, NAD1, and NAD2. Nevertheless, intraspecific divergence remained within the range typically observed for mitochondrial protein-coding genes in closely related taxa.

Patterns of interspecific genetic distances were concordant with PCA structure. Pairwise distances between *M. spinolai* and the other species ranged from 0.032 to 0.037 (NAD6) to 0.060–0.160 (ATP8) depending on the gene, with most loci falling between 0.050 and 0.090. Distances between *M. gajardoi* and *M. parapatrica* ranged from 0.030 to 0.045 (NAD6) to 0.040–0.160 (ATP8) across genes, with the majority of loci clustering between 0.070 and 0.100. The highest values (up to 0.160) were observed in more variable genes, particularly ATP8.

Overall, both PCA and K2P distance analyses consistently support clear species-level differentiation among the three *Mepraia* taxa, while also revealing heterogeneous patterns of intraspecific variability across loci, particularly in shorter and more rapidly evolving mitochondrial genes.

Analysis of selective pressure on mitochondrial protein-coding genes revealed heterogeneous selection profiles across loci, indicating that selective signals are unevenly distributed among genes (Table 3). The strongest evidence of positive selection was detected in NAD5, NAD6, NAD1, and COIII, which together concentrated the majority of candidate sites identified across all analyses.

**Table 3:** Summary of positive selection analysis on *Mepraia* mitochondrial protein-coding genes

Gene	MEME (sites)	FUBAR (sites)	BUSTED (p-value)
NAD5	17	37	0.5000
NAD6	5	10	0.5000
NAD1	5	10	0.5000
COIII	2	9	0.5000
COII	0	1	0.5000
NAD4L	1	0	0.5000
ATP6	1	0	0.5000
NAD4	1	0	0.5000
CytB	0	0	0.5000
ATP8	1	0	0.5000
NAD2	1	0	0.5000
NAD3	1	0	0.5000
COI	1	0	0.5000

NAD5 exhibited the strongest signal, with 17 sites detected by MEME and 37 by FUBAR. NAD5 showed the highest number of candidate sites, with 17 detected by MEME and 37 by FUBAR. Similarly, NAD6 and NAD1 each showed five sites under episodic selection and ten under pervasive selection. The COIII gene displayed fewer candidate sites overall, with two sites identified by MEME and nine by FUBAR.

Despite the presence of multiple site-level signals detected by MEME and FUBAR, BUSTED analyses did not provide support for gene-wide positive selection in any of these loci (*p* = 0.5000), indicating that positive selection is restricted to specific codons rather than acting across entire genes.

The remaining mitochondrial genes (COII, NAD4L, ATP6, NAD4, ATP8, NAD2, NAD3, and COI) showed limited evidence of positive selection. For each of these genes, MEME identified only a single candidate site under episodic selection, while FUBAR detected no sites under pervasive selection, with the exception of COII, which exhibited one site exceeding the posterior probability threshold. Consistently, BUSTED analyses for all these loci were non-significant (p = 0.5000), supporting the absence of gene-wide selection signals. No evidence of positive selection was detected for CytB by any method (MEME, FUBAR, or BUSTED), indicating strong evolutionary constraint on this gene in the analyzed dataset.

## Discussion

Phylogenetic and evolutionary analyses based on transcriptome-derived mitochondrial genomes provide a robust framework for interpreting genetic diversity and lineage relationships in this endemic Chilean lineage of Triatominae. As key vectors of *T. cruzi*, the etiological agent of Chagas disease, these insects remain comparatively understudied despite their epidemiological importance (Ramírez et al. 2026). The genus *Mepraia* comprises three species with distinct ecological and geographic distributions, and reconstructing their evolutionary history is essential for refining taxonomy and informing vector biology (Chacón et al. 2016; Frías-Lasserre et al. 2018; Garrido et al. 2019).

Partial mitochondrial genomes of *M. gajardoi*, *M. parapatrica*, and *M. spinolai* enabled the recovery of a complete and colinear set of the 13 mtDNA PCGs. The conserved gene order, consistent with that of *T. infestans* (Pita et al. 2017), supports the structural stability of triatomine mitogenomes. Variation in tRNA recovery is most parsimoniously explained by differences in transcript representation rather than genomic rearrangement. Additionally, AT content values were consistent with previously reported patterns in Triatominae and other insects (Cigarroa-Toledo et al. 2024; Almeida et al. 2025; Behura 2015; Desalle 2017), supporting the reliability of the reconstructed sequences for downstream evolutionary analyses.

The mtDNA PCGs are particularly relevant due to their extensive use in phylogenetic inference (Justi et al. 2014; Belintani et al. 2020; Sun et al. 2022; Silva et al. 2023; Groppo et al. 2025) and their widespread application in molecular identification of insect vectors through DNA barcoding approaches (Hebert et al. 2003; Cywinska et al. 2006; Monteiro et al. 2004). The strong phylogenetic resolution obtained in this study empirically supports the robustness of transcriptome-derived mitochondrial assemblies for evolutionary analyses in Triatominae. Although transcriptome-based approaches may underrepresent non-coding regions, coding sequences typically show high concordance with DNA-derived mitogenomes (Nabholz et al. 2010; Forni et al. 2019.

Moreover, the topological congruence observed between our results and previous mitogenomic studies (Dotson and Beard 2001; Pita et al. 2017), as well as with whole-genome data from *Triatoma mexicana* (Herrich-Schaeffer, 1848) (Aguilera-Uribe et al. 2020) and *Panstrongylus geniculatus* (Latreille 1811) (Canizales-Silva et al. 2025), further supports the biological accuracy of the reconstructed sequences and supports the reliability of transcriptome-derived mitochondrial data for recovering evolutionary patterns in Triatominae. Within this framework, the sampling design is consistent with the objectives of reconstructing mitochondrial genes from RNA-Seq data. It is also appropriate for evaluating species-level evolutionary relationships (Belintani et al. 2023). Because mitochondrial genomes are haploid and maternally inherited, a moderate number of individuals is sufficient to recover species-level lineages and conserved gene organization patterns, as reflected in the well-resolved phylogenetic structure obtained (Ballard and Whitlock 2004). Although this design was not intended to exhaustively characterize intraspecific diversity, the dataset provides a robust structure for phylogenetic inference and comparative evolutionary analyses.

Transcriptome-derived mitochondrial datasets provide a reliable and efficient resource for evolutionary analyses in non-model insects. Because mitochondrial transcripts are often abundant and expressed as polycistronic units, RNA-Seq data can yield high coverage across coding regions, enabling robust recovery of mitochondrial protein-coding genes without additional targeted sequencing (Smith 2013; Tian and Smith 2016; Hahn et al. 2013). In our study, all 13 mitochondrial protein-coding genes were consistently recovered across samples, and the resulting phylogenetic patterns were highly congruent with previous mitochondrial and nuclear-based hypotheses for Triatominae. Together, these results support the reliability of transcriptome-derived mitochondrial coding sequences for phylogenetic inference in *Mepraia*, consistent with previous studies demonstrating strong concordance between RNA-derived and DNA-derived mitogenomes (Nabholz et al. 2010; Forni et al. 2019).

Nevertheless, transcriptome-based assemblies have important limitations. Lowly expressed regions, particularly some tRNAs and the control region, may be incompletely recovered, which restricts the reconstruction of complete mitochondrial genomes (Tian and Smith 2016; Forni et al. 2019). For this reason, the variation observed here in tRNA recovery is more plausibly explained by differences in transcript representation rather than by true structural differences among mitogenomes. In addition, transcriptomic data alone do not allow definitive inference of complete genome architecture without complementary DNA-based validation (Hahn et al. 2013). Accordingly, the present dataset should be interpreted primarily as a robust mitochondrial coding resource for phylogenetic and molecular evolutionary analyses rather than as a complete structural characterization of the mitogenome.

Phylogenetic analyses based on the concatenation of the 13 mtDNA PCGs recovered the three *Mepraia* species as reciprocally monophyletic and strongly supported clades. *M. parapatrica* and *M. gajardoi* were consistently inferred as sister taxa, whereas *M. spinolai* was positioned as the earliest diverging lineage within the genus. This topology corroborates previous hypotheses based on both mitochondrial and nuclear markers (Campos-Soto et al. 2015, 2020) and agrees with recent transcriptome-based inferences (Belintani et al. 2023), reinforcing the evolutionary distinctiveness of *M. spinolai*. The consistency of these relationships across inference methods, together with their agreement with previous multilocus studies (Justi et al. 2014; Kieran et al., 2021; Masonick et al. 2025) and the conserved gene order and composition observed here, supports the robustness of the mitochondrial coding dataset and the reliability of transcriptome-derived assemblies for resolving recent divergences within Triatominae.

Thus, the pattern observed in the comprehensive phylogeny with 69 taxa of Triatominae is consistent and demonstrates that mitogenomes provide sufficient resolution for recovering shallow and moderately deep divergences within Triatominae. The resolution obtained here contrasts with studies based on a limited number of isolated markers, which often produce reduced node support or incongruent topologies in Triatominae (Justi et al. 2014) or other groups (Kwon et al. 2012; Zarrei et al. 2015). Complete mitogenomes and extended multilocus data generally provide stronger phylogenetic signal and more reliable inference of species relationships than single gene fragments (Fraumene et al. 2006; Finnegan et al. 2025).

Furthermore, the topology recovered for *Mepraia* in the comprehensive analysis is consistent with phylogenomic inferences based on ultraconserved elements (UCEs) (Kieran et al. 2021) and with multilocus nuclear datasets (Belintani et al. 2023). Although UCE-based studies emphasize broader sampling across Triatominae and include lower intrageneric representation within *Mepraia*, the relative position of the Chilean lineages remains congruent with the general structure observed in nuclear analyses. This concordance among mitochondrial, transcriptomic, and UCE-derived datasets indicates that the phylogenetic signal in *Mepraia* is not solely attributable to mitochondrial inheritance.

At a broader scale within Triatominae, the topology inferred from the concatenated 13 PCGs shows strong agreement with multilocus and phylogenomic hypotheses (Justi 2014; Justi et al. 2016; Kieran et al. 2021; Masonick et al. 2025). In the comprehensive analysis, *Mepraia* was recovered within the South American *Triatoma* radiation, forming a sister relationship with the clade comprising Central and North American species. This placement mirrors the geographic structuring recovered using thousands of UCE loci (Kieran et al. 2021), in which major regional clades within Triatomini are consistently resolved. Similarly, the recovery of monophyletic clades corresponding to *Rhodnius*, *Panstrongylus*, *Paratriatoma*, and *Hospesneotomae* is consistent with previous phylogenetic studies based on mitochondrial and nuclear markers (Justi et al. 2014; Kieran et al. 2021; Paiva et al. 2025), reinforcing the stability of these generic lineages.

Some incongruences were restricted to deeper nodes, particularly in the relationships among major geographic lineages. These patterns are consistent with known limitations of mitochondrial datasets, in which substitutional saturation and compositional heterogeneity may compromise phylogenetic resolution at older evolutionary scales (Cameron 2014; Justi et al. 2014). In contrast, multilocus nuclear datasets, especially those comprising hundreds to thousands of loci, have demonstrated greater stability at basal nodes in Triatominae (Kieran et al. 2021; Filée et al. 2022) and across diverse animal groups (Lemmon et al. 2012; Cannon and Kocot 2016; Foley et al. 2019).

Despite these limitations, the strong topological congruence between the topology recovered here and previously published nuclear phylogenomic frameworks supports the reliability of complete mitochondrial coding datasets as an efficient tool for phylogenetic reconstruction in Triatominae, particularly at species-level and intrageneric scales.

Based on the well-established phylogenetic structure recovered here, genetic distance and PCA analyses further reinforce the distinction among the three *Mepraia* species, while revealing contrasting patterns of intraspecific variability. Previous phylogeographic studies demonstrated strong population structuring within the genus, identifying three lineages congruent with the currently recognized species (Campos-Soto et al. 2013). More recent analyses using single nucleotide polymorphisms detected subtle structuring within *M. spinolai*, including differences in genetic diversity between central and peripheral populations (San Juan et al. 2021).

Our estimates indicate that *M. spinolai* exhibits consistently low intraspecific genetic distances and forms a compact cluster in PCA space. This pattern aligns with earlier population genetic studies suggesting relatively high connectivity and limited genetic subdivision across its distribution range. The low mitochondrial divergence observed in *M. spinolai* contrasts with the structuring detected using nuclear SNPs (San Juan et al. 2021), indicating potential discordance between mitochondrial and nuclear histories. Such discordance may reflect demographic processes, such as recent population expansions, that homogenized the maternal lineage (Avise 2000).

In contrast, *M. parapatrica* and, to a lesser extent, *M. gajardoi* exhibit higher intraspecific variability, particularly in rapidly evolving mitochondrial genes such as ATP8. *M. gajardoi* shows moderate and locus-specific variability, with most genes displaying low divergence and higher values restricted to faster-evolving loci such as ATP8 and NAD3. *M. parapatrica* exhibits comparatively greater intraspecific dispersion for certain loci, particularly ATP8 and NAD3, while most other genes remain comparatively conserved. Their broader dispersion in PCA space and the gene-specific distance patterns detected in ATP8, NAD3, and ATP6 are consistent with patterns of strong population subdivision previously reported for *Mepraia* populations (Campos-Soto et al. 2015, 2020, 2022). This pattern is also consistent with the K2P distance ranges, which show elevated maximum values in these loci compared to more conserved genes. These studies identified pronounced geographic structuring across the distribution range of the genus, suggesting that long-term demographic subdivision may contribute to the accumulation of mitochondrial variation within species. Phylogenomic studies based on transcriptomes have further shown that *Mepraia* underwent rapid diversification and ancient introgression events, resulting in reticulated phylogenetic patterns and overall low genetic diversity (Belintani et al. 2023). Within this framework, the elevated variability observed in *M. parapatrica* likely reflects a more structured demographic history and the accumulation of divergence in fast-evolving loci, consistent with the retention of locus-specific evolutionary signals potentially associated with introgression.

The markedly elevated intraspecific divergence observed for ATP8 in *M. parapatrica* merits specific consideration. Technical artefacts are unlikely, as sequencing coverage was high, open reading frames were intact, and the signal was restricted to ATP8 rather than affecting multiple loci. A more parsimonious explanation relates to the intrinsic properties of ATP8, one of the shortest and fastest-evolving mitochondrial protein-coding genes, characterized by relaxed functional constraints and elevated substitution rates in insects (Boore 1999; Dong et al. 2021). In lineages experiencing prolonged population structure and isolation, these characteristics may promote locus-specific divergence (Avise et al. 1987). This interpretation is further supported by previous evidence of long-term population structuring within *Mepraia* (Campos-Soto et al. 2015, 2020) and by documented ancient introgression events (Belintani et al. 2023). Accordingly, the high intraspecific variation detected here most likely represents genuine biological signal rather than methodological bias.

Differences relative to earlier population-level studies are primarily attributable to variation in marker choice and genomic resolution. Whereas previous analyses focused on demographic patterns using a limited number of mitochondrial fragments or nuclear markers, the present study examines variation at the mitogenomic scale across all 13 mitochondrial PCGs. This broader genomic perspective allows the detection of heterogeneity among individual mitochondrial loci, revealing that intraspecific variability in *M. parapatrica* is unevenly distributed across the mitogenome. Consequently, loci with faster evolutionary rates, such as ATP8 and NAD3, contribute disproportionately to the overall signal of intraspecific divergence detected here, whereas more conserved genes such as COI, NAD1, and NAD2 retain comparatively low levels of variation.

Selection analyses revealed heterogeneous evolutionary pressures across mitochondrial PCGs. Site-level approaches (FUBAR and MEME) identified candidate codons under positive selection in NAD5, NAD6, NAD1, and COIII, whereas most other genes showed little evidence of diversifying selection. In contrast, BUSTED did not detect gene-wide episodic positive selection in any locus, indicating that adaptive signals are localized to specific codons rather than acting across entire genes. This pattern is consistent with expectations for mitochondrial genomes, which are typically subject to strong purifying selection due to their central role in oxidative phosphorylation (Ballard and Whitlock 2004; Rand et al. 2004). Empirical studies have shown that adaptive evolution in mitochondrial genes is usually confined to discrete functional sites rather than occurring gene-wide (Meiklejohn et al. 2007; Fonseca et al. 2008).

At the pathway level, genes showing the strongest selection signals encode subunits of Complex I (NAD1, NAD5, NAD6) and Complex IV (COIII), suggesting that selective pressures may preferentially affect components of the oxidative phosphorylation machinery. Complex I, in particular, has repeatedly been implicated in adaptive mitochondrial evolution across animal taxa (Mishmar et al. 2003; Murray and Horscroft 2016). However, in the absence of structural modeling or experimental validation, functional consequences cannot be inferred, and selection signals are interpreted conservatively as evidence of localized adaptive divergence amid pervasive purifying selection.

In the absence of structural modeling or experimental validation, functional effects cannot be confidently attributed to individual amino acid substitutions. Accordingly, signals of selection are interpreted conservatively, supported by concordant patterns across independent analyses, including genetic distances and PCA. Overall, these results are consistent with localized adaptive divergence occurring within a background of strong purifying selection, with fine-scale adaptive processes restricted to specific codons.

These molecular patterns provide additional context for existing evolutionary hypotheses proposed for the genus. The coexistence of localized positive selection signals and heterogeneous intraspecific diversity aligns with scenarios of historical population subdivision and lineage diversification, including the rapid diversification and ancient hybridization framework suggested by Belintani et al. (2023). Specifically, the elevated variability in *M. parapatrica* is consistent with biogeographic models invoking isolation and dispersal between insular and continental populations (Campos-Soto et al. 2022). Furthermore, the combination of strong purifying selection with codon-specific adaptive signals fits models of divergence under partial reproductive isolation, supported by cytogenetic differentiation (Frías-Lasserre 2010) and experimental evidence of both pre- and post-zygotic barriers within the genus (Campos-Soto et al. 2016).

By integrating phylogenetic inference, genetic distance analyses, and codon-level selection tests, this study provides a comprehensive view of lineage divergence and evolutionary dynamics in *Mepraia*. The combined evidence supports a refined understanding of species boundaries and is consistent with previously proposed scenarios of population structuring, introgression, and diversification within the genus (Campos-Soto et al. 2016, 2022; Belintani et al. 2023). Patterns of intraspecific variability across mitochondrial loci further highlight the importance of genome-wide approaches for capturing heterogeneous evolutionary signals. In this context, the interplay between purifying selection and localized adaptive processes is consistent with models of divergence under partial reproductive isolation, as suggested by cytogenetic and experimental studies (Frías-Lasserre 2010; Campos-Soto et al. 2016). Collectively, these findings provide a framework for future integrative studies combining genomic, ecological, and functional approaches, and establish a mitogenomic reference for population genetics, phylogeography, and comparative analyses of Triatominae vectors.

In conclusion, the transcriptome-derived mitochondrial genomes analyzed here provide robust evidence for the monophyly and internal relationships of *Mepraia*, while also revealing heterogeneous intraspecific variation and localized signals of adaptive evolution within a background of strong purifying selection. Beyond clarifying evolutionary patterns in an endemic and epidemiologically relevant triatomine lineage, our results demonstrate that publicly available RNA-Seq data can be effectively used to recover mitochondrial coding datasets in non-model insects. These findings expand the genomic resources available for Triatominae and provide a comparative framework for future studies integrating mitochondrial, nuclear, ecological, and functional data to better understand diversification and vector biology in this group.

## Supplementary Information

Below is the link to the electronic supplementary material.

Supplementary file1 (PDF 225 KB)

Supplementary file2 (XLSX 30 KB)

Supplementary file3 (PDF 165 KB)

Supplementary file4 (PDF 445 KB)
