# Supplementary material for: Mitochondrial genomes from RNA-Seq reveal phylogeny and selection in Mepraia (Hemiptera: Reduviidae)
Source: Mol Genet Genomics. 2026 May 19;301(1):117. doi: 10.1007/s00438-026-02434-y (PMC13186837; doi:10.1007/s00438-026-02434-y)

Figure S1. Principal Component Analysis (PCA) based on nucleotide variation in the *ATP6* gene across *Mepraia* species.

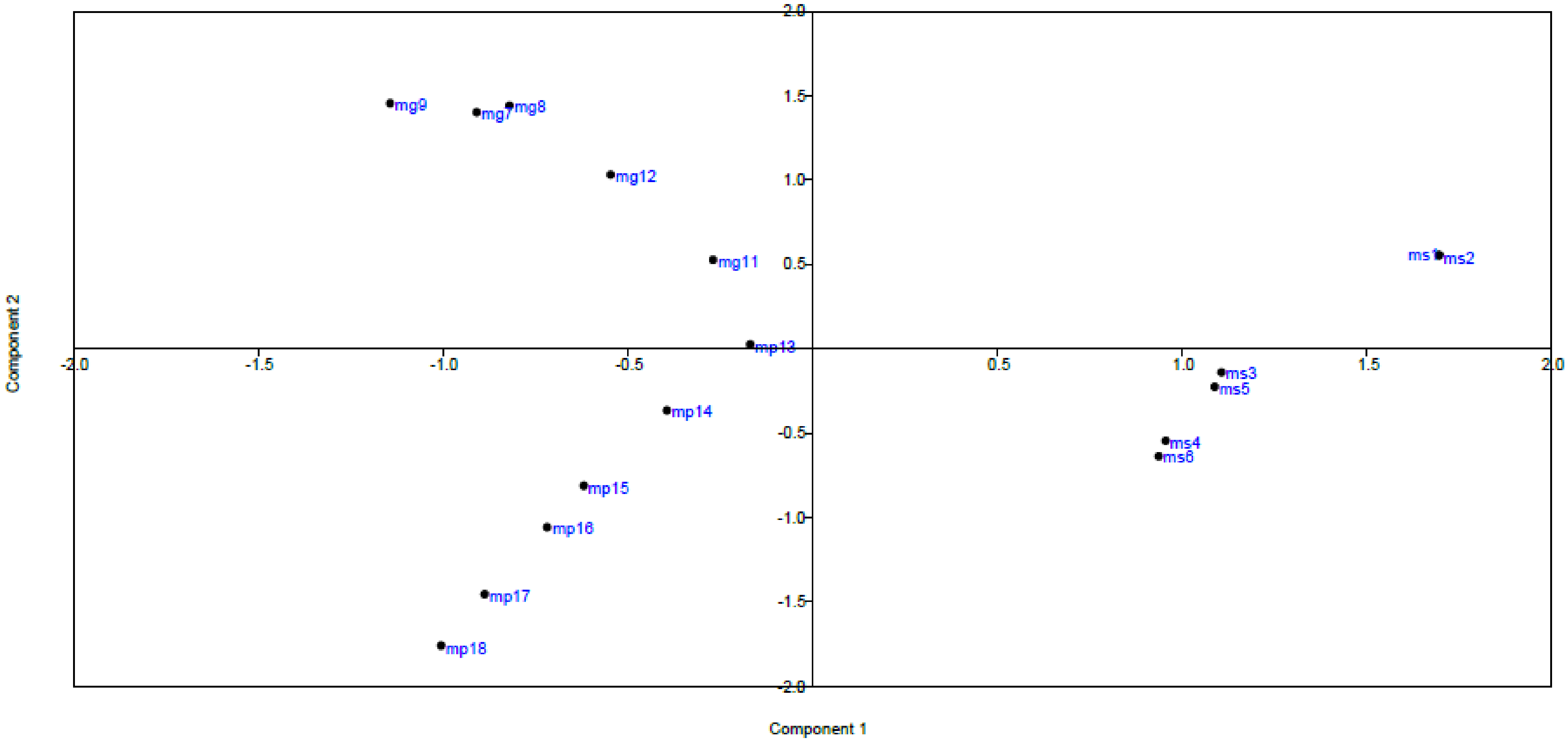

Figure S2. Principal Component Analysis (PCA) based on nucleotide variation in the *ATP8* gene across *Mepraia* species.

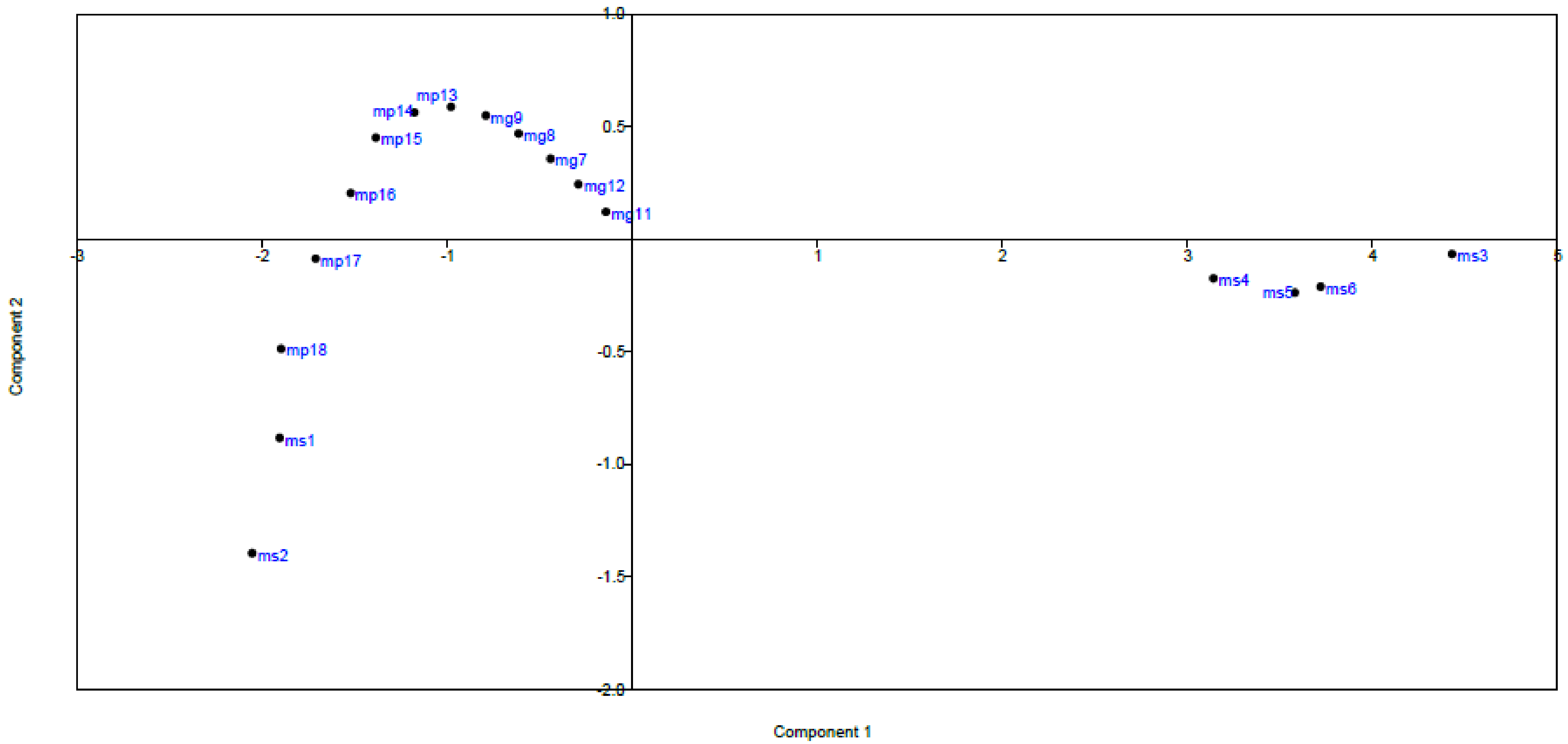

Figure S3. Principal Component Analysis (PCA) based on nucleotide variation in the *COI* gene across *Mepraia* species.

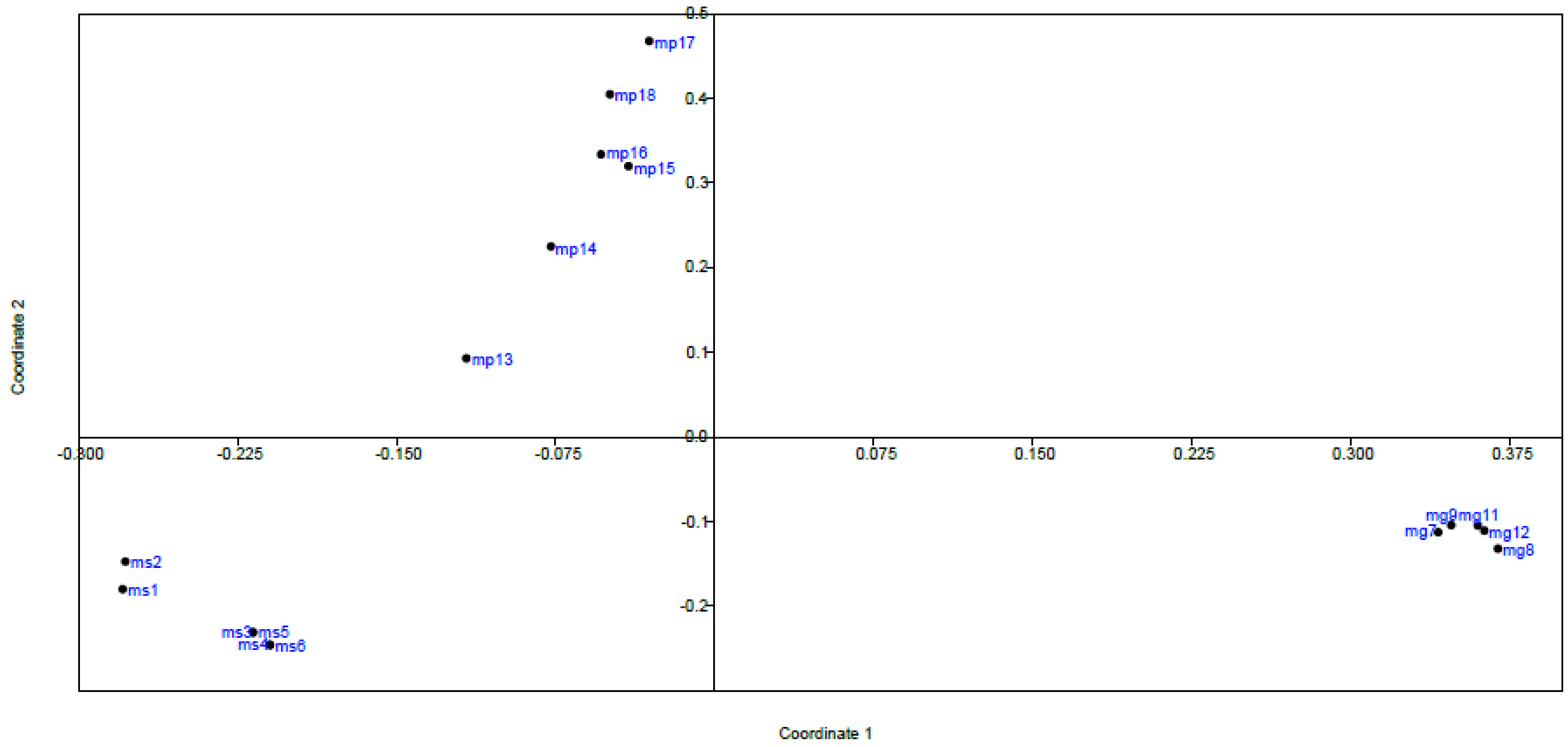

Figure S4. Principal Component Analysis (PCA) based on nucleotide variation in the *COII* gene across *Mepraia* species.

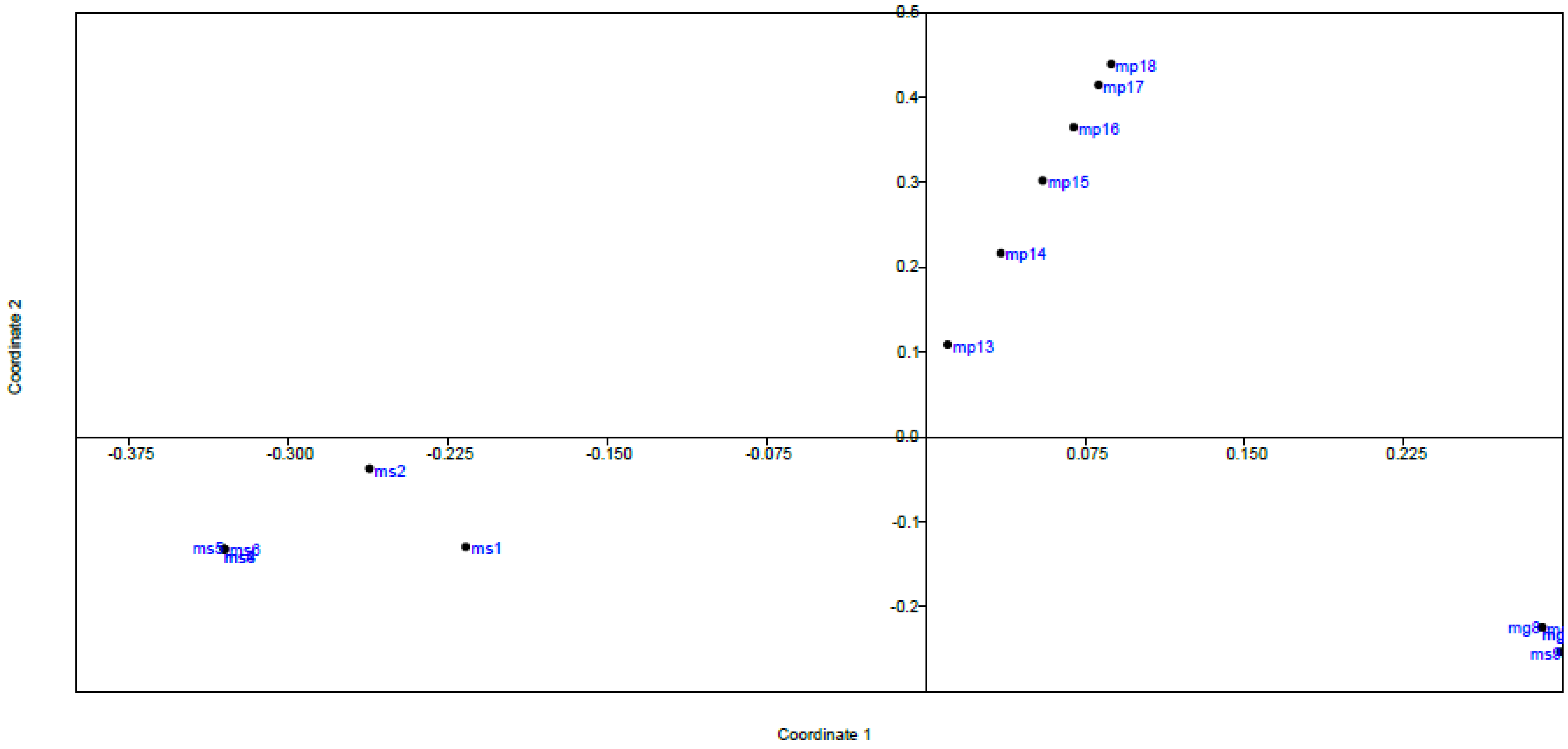

Figure S5. Principal Component Analysis (PCA) based on nucleotide variation in the *COIII* gene across *Mepraia* species.

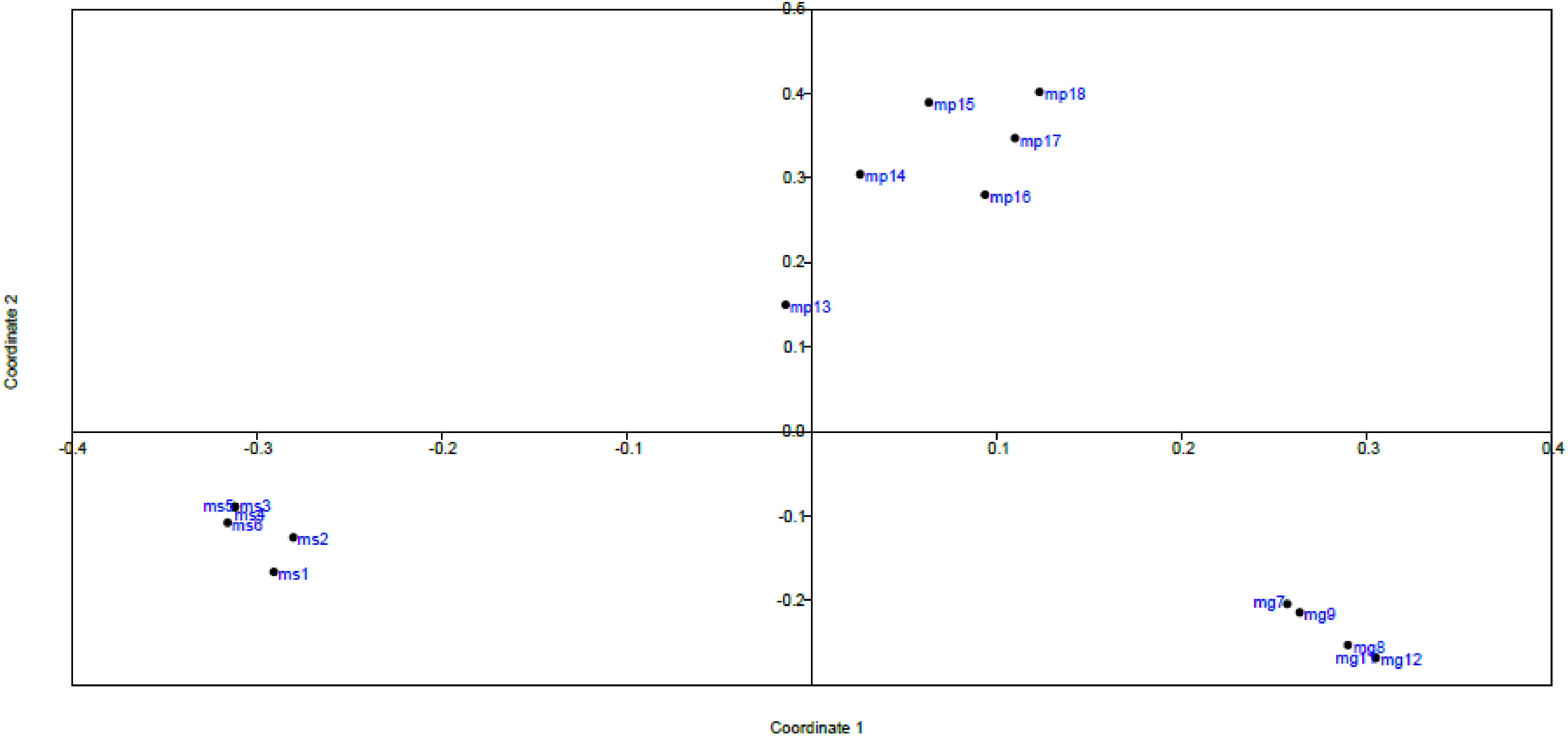

Figure S6. Principal Component Analysis (PCA) based on nucleotide variation in the *CYTB* gene across *Mepraia* species.

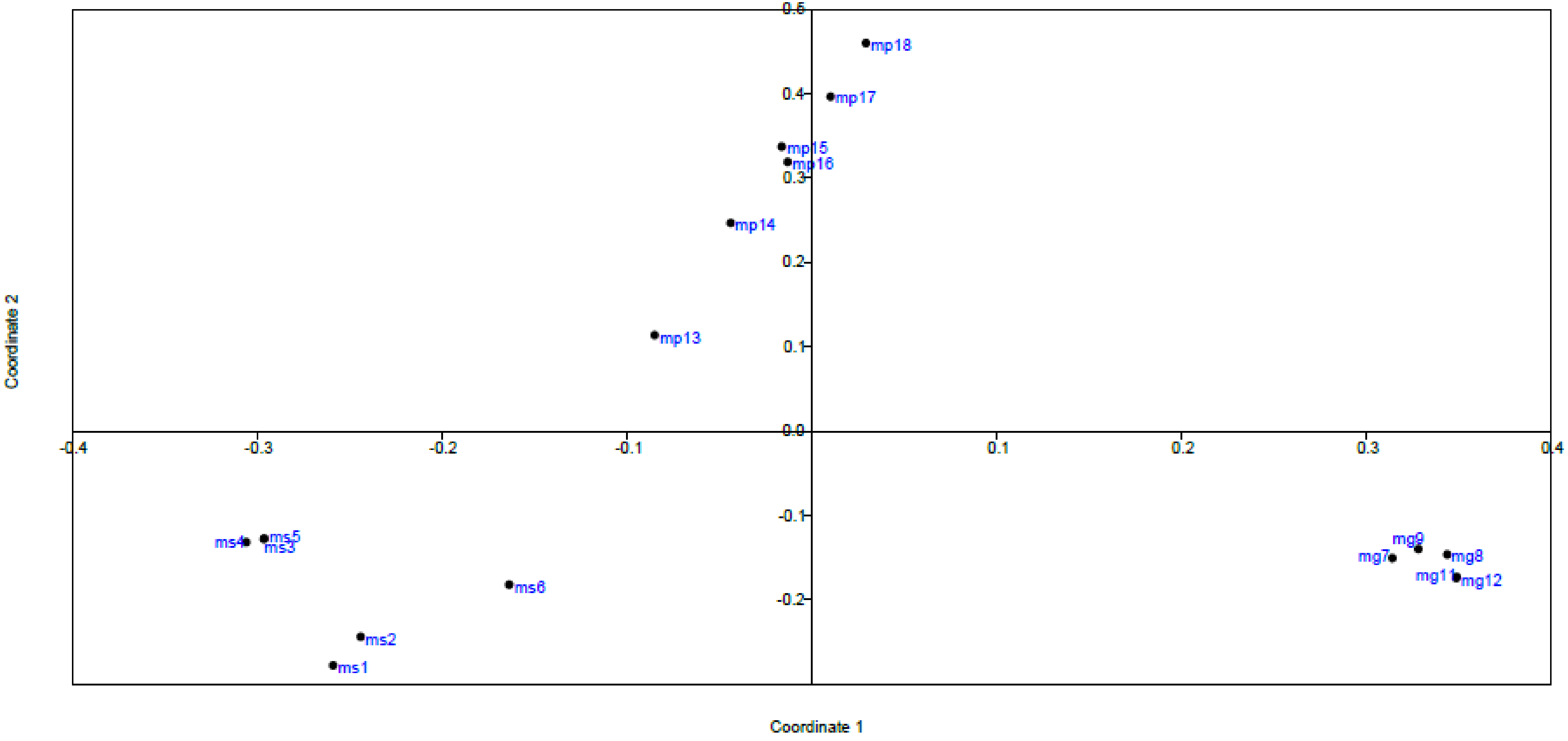

Figure S7. Principal Component Analysis (PCA) based on nucleotide variation in the *NAD1* gene across *Mepraia* species.

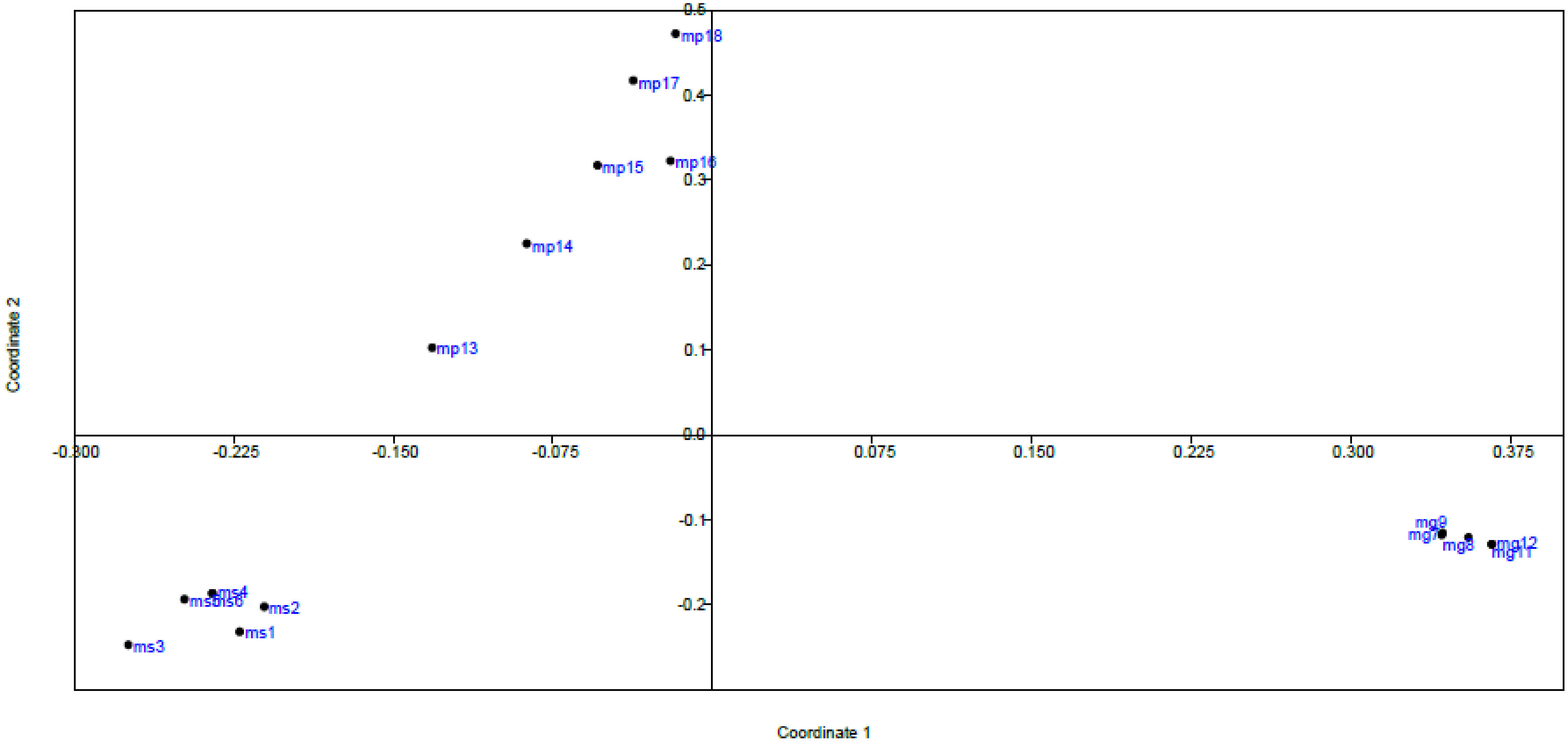

Figure S8. Principal Component Analysis (PCA) based on nucleotide variation in the *NAD2* gene across *Mepraia* species.

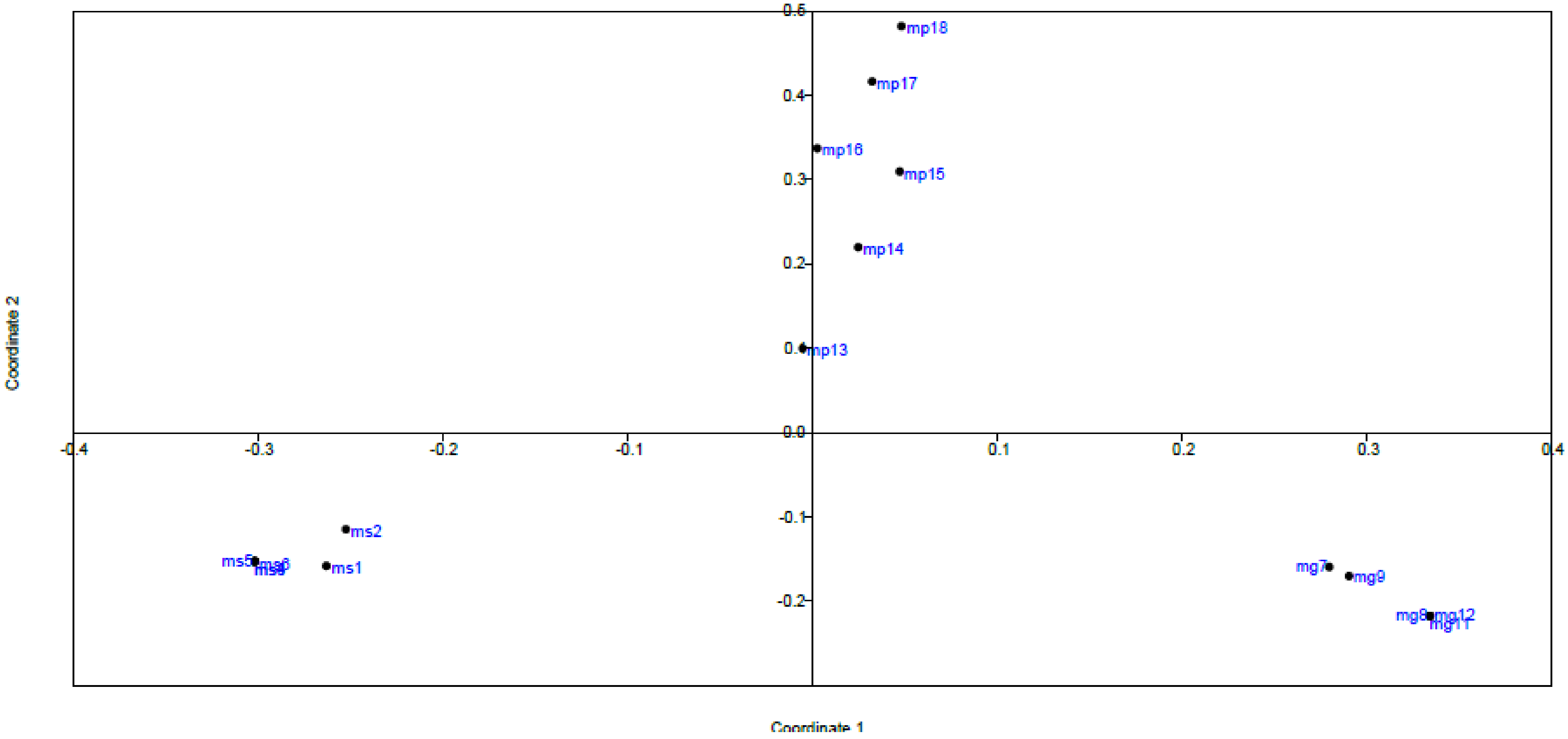

Figure S9. Principal Component Analysis (PCA) based on nucleotide variation in the *NAD3* gene across *Mepraia* species.

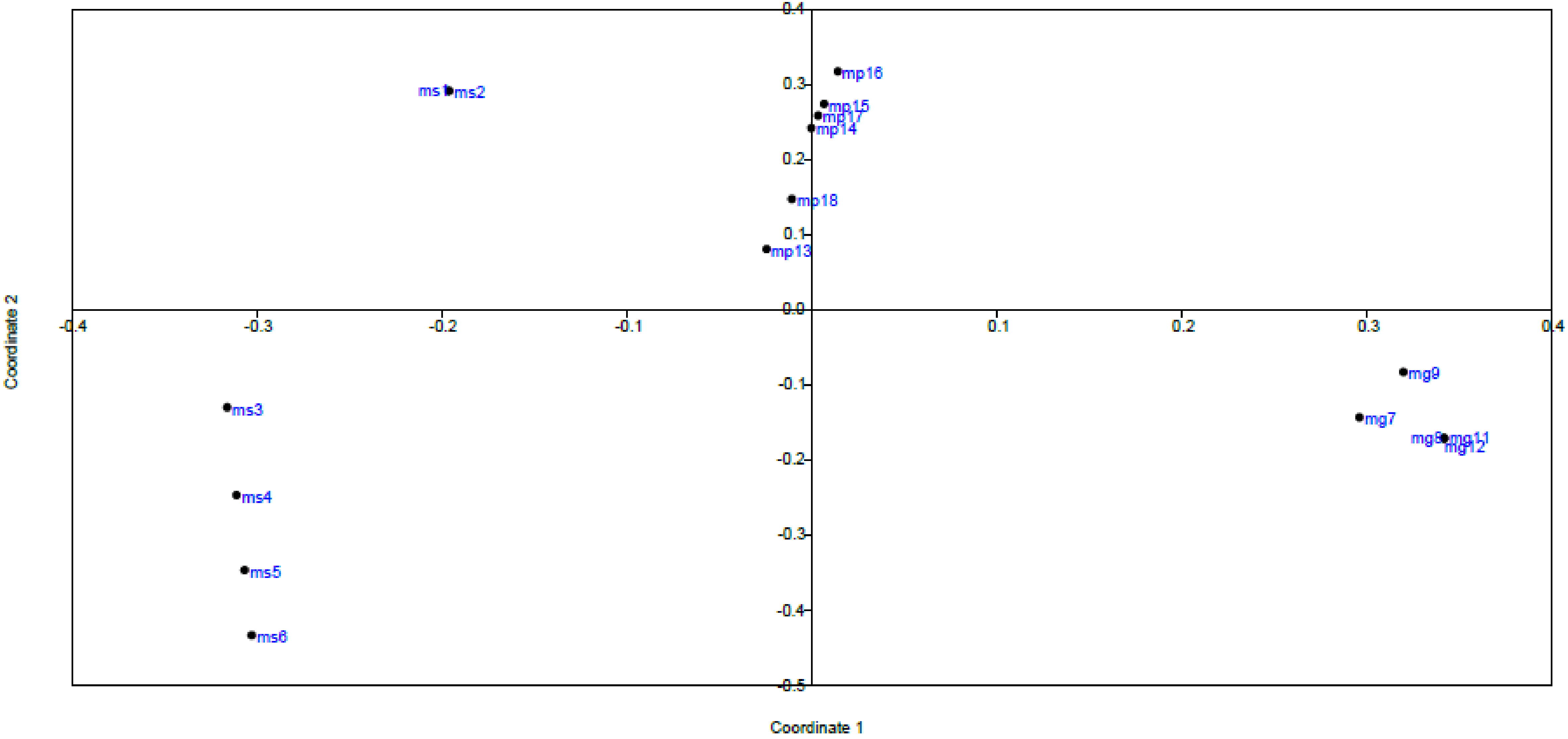

Figure S10. Principal Component Analysis (PCA) based on nucleotide variation in the *NAD4* gene across *Mepraia* species.

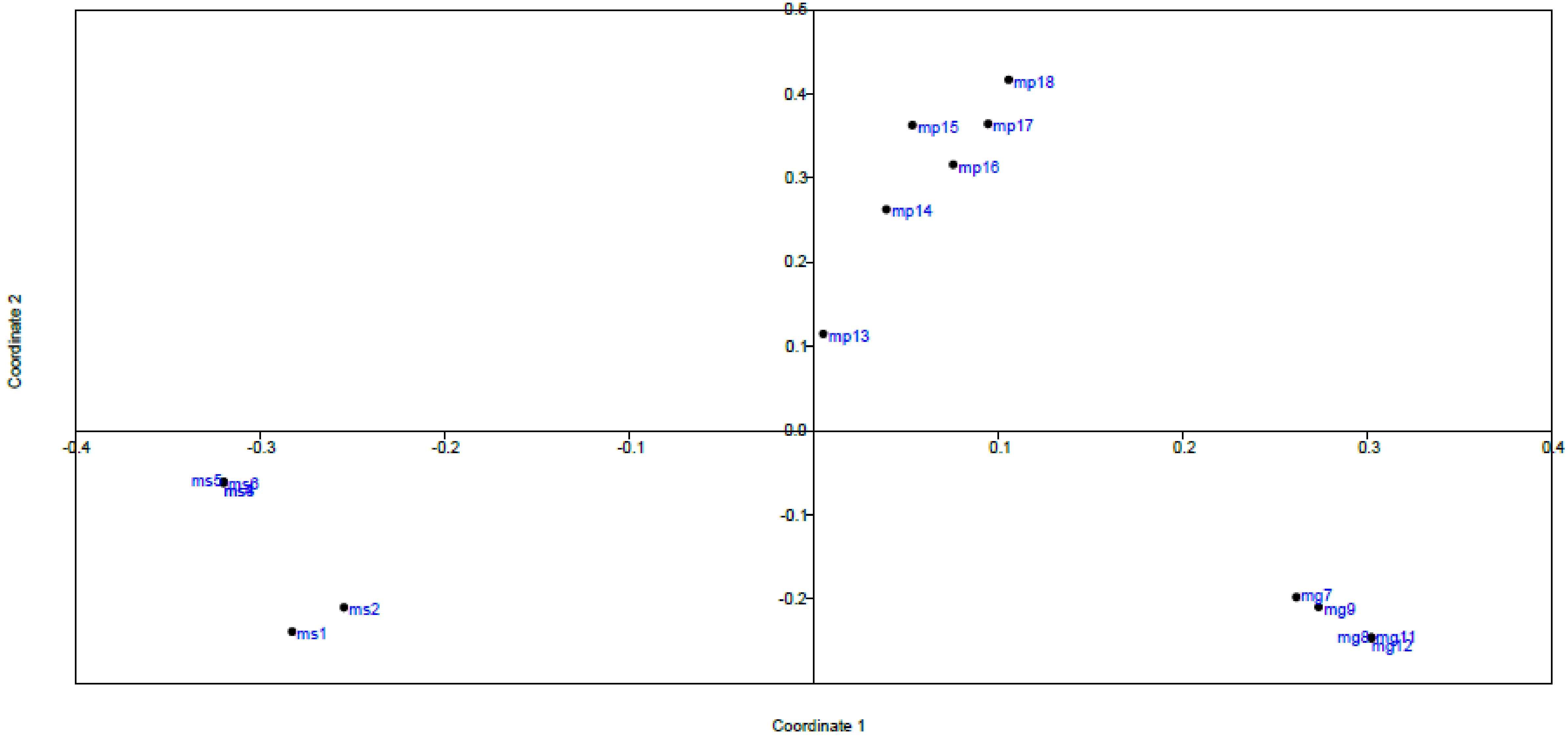

Figure S11. Principal Component Analysis (PCA) based on nucleotide variation in the *NAD4L* gene across *Mepraia* species.

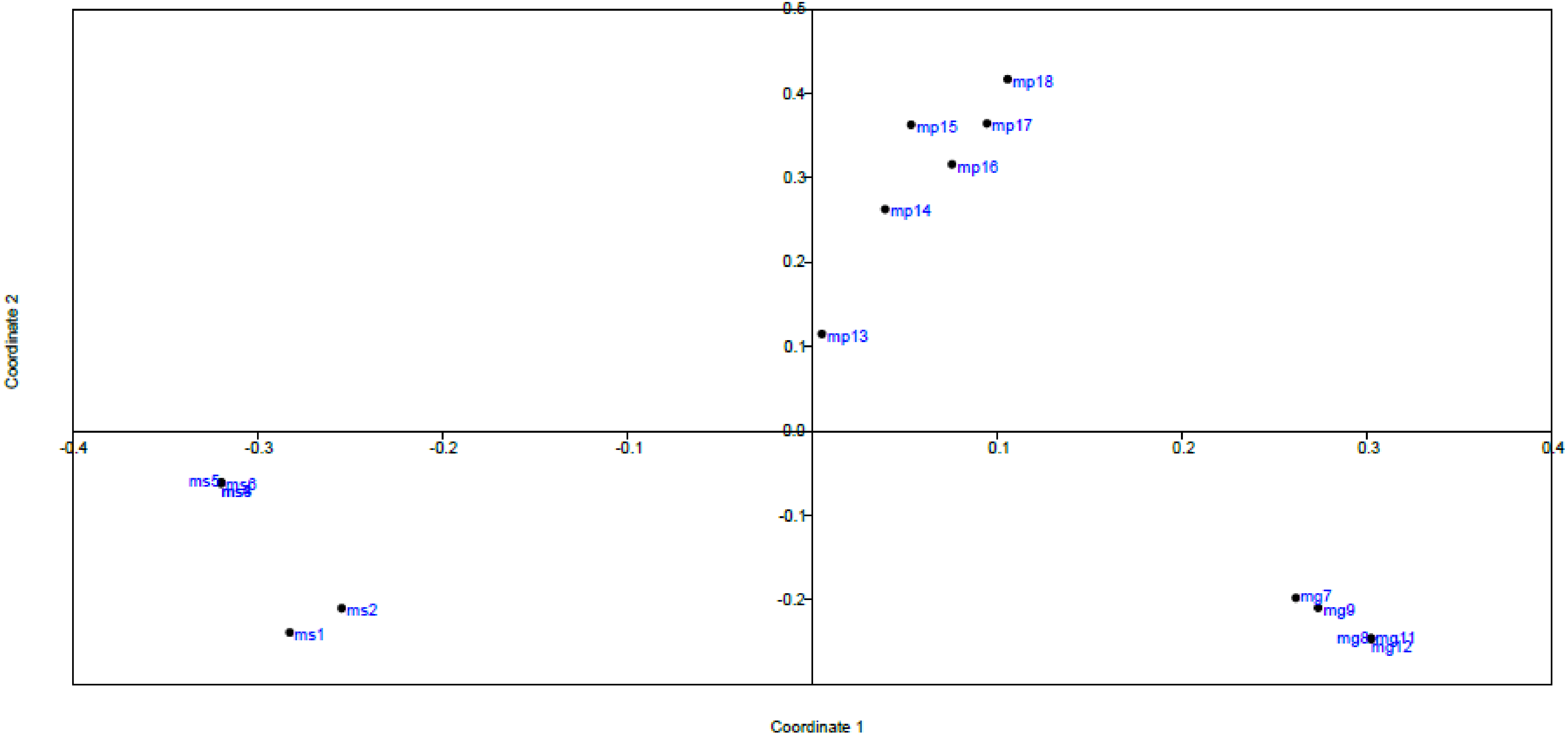

Figure S12. Principal Component Analysis (PCA) based on nucleotide variation in the *NAD5* gene across *Mepraia* species.

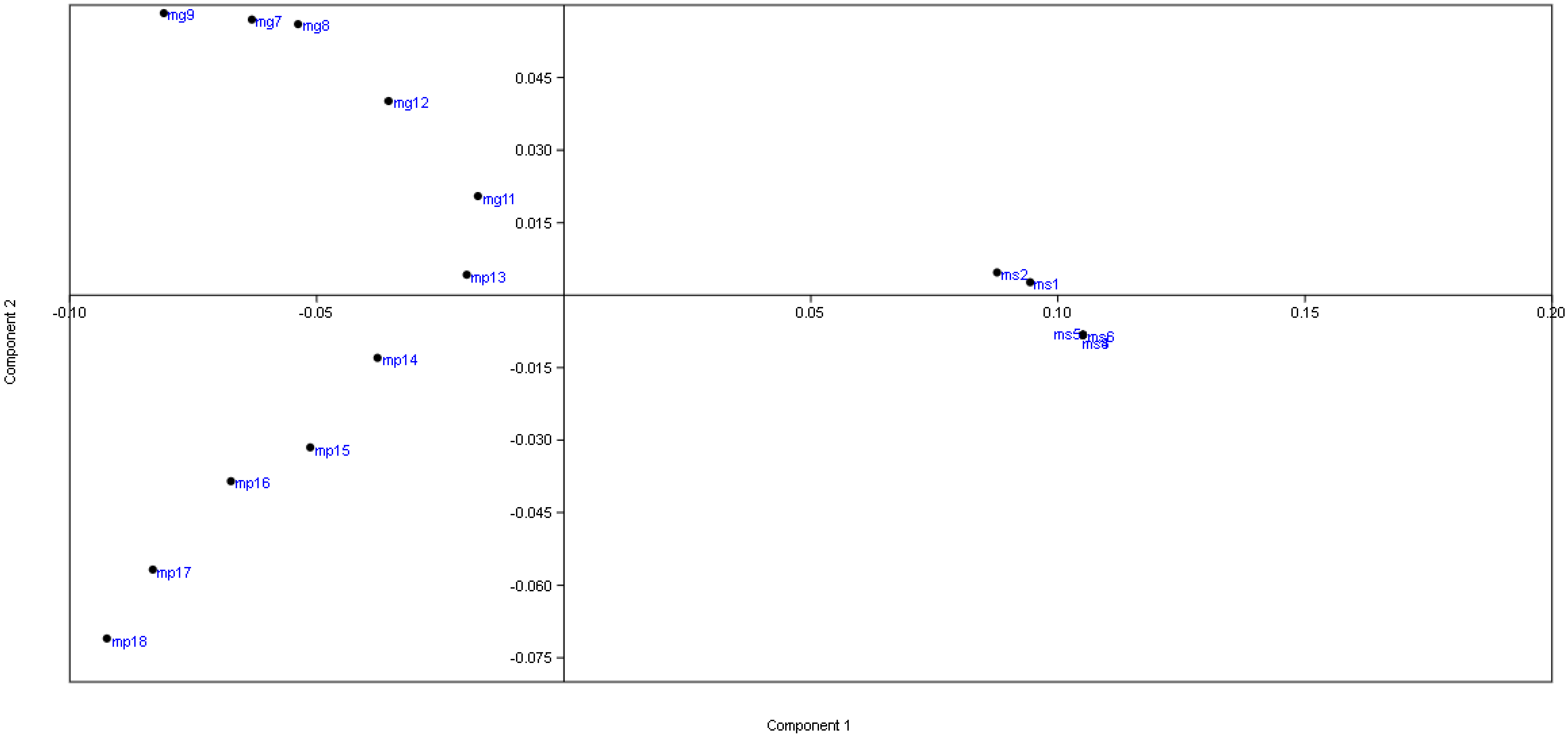

Figure S13. Principal Component Analysis (PCA) based on nucleotide variation in the *NAD6* gene across *Mepraia* species.

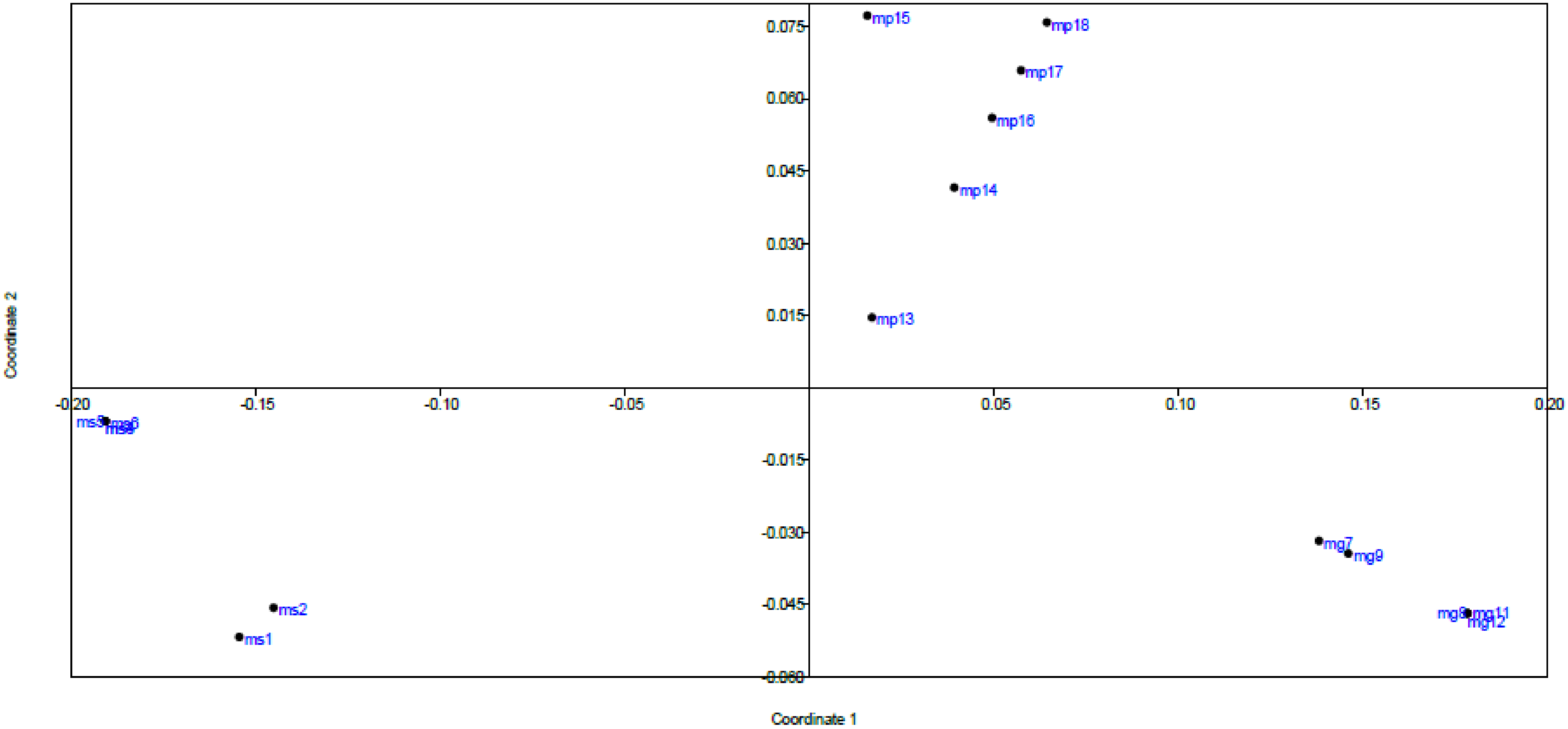

Supplement: Supplementary file 1 — Supplementary file1 (PDF 225 KB) [file 438_2026_2434_MOESM1_ESM.pdf]
