# Supplementary material for: Mitochondrial genomes from RNA-Seq reveal phylogeny and selection in Mepraia (Hemiptera: Reduviidae)
Source: Mol Genet Genomics. 2026 May 19;301(1):117. doi: 10.1007/s00438-026-02434-y (PMC13186837; doi:10.1007/s00438-026-02434-y)
Supplement: Supplementary file 3 — Supplementary file3 (PDF 165 KB) [file 438_2026_2434_MOESM3_ESM.pdf]

Table S1. Specimens used in the study and SRA accession code on GenBank.

IDs	Species	Acession Code
Ms1	*M. spinolai*	SRR23005999
Ms2	*M. spinolai*	SRR23005996
Ms3	*M. spinolai*	SRR23006000
Ms4	*M. spinolai*	SRR23005995
Ms5	*M. spinolai*	SRR23006001
Ms6	*M. spinolai*	SRR23005994
Mg7	*M. gajardoi*	SRR23006003
Mg8	*M. gajardoi*	SRR23006002
Mg9	*M. gajardoi*	SRR23005988
Mg10	*M. gajardoi*	SRR23005990
Mg11	*M. gajardoi*	SRR23005988
Mg12	*M. gajardoi*	SRR23005991
Mp13	*M. parapatrica*	SRR23005987
Mp14	*M. parapatrica*	SRR23005992
Mp15	*M. parapatrica*	SRR23005986
Mp16	*M. parapatrica*	SRR23005993
Mp17	*M. parapatrica*	SRR23005998
Mp18	*M. parapatrica*	SRR23005997

Table S2. Mitochondrial genome data from triatomine species

Species	Genome Size (bp)	Accession Code
Triatoma huehuetenanguensis	17,832	OP345451.1
Triatoma sp.	15,842	OP345450.1
Triatoma sp.	15,772	OP345449.1
Triatoma dimidiata	15,916	OP345448.1
Triatoma sp.	15,716	OP345447.1
Triatoma sanguisuga	15,542	CM085471.1
Triatoma boliviana	16,719	NC_086924.1
Triatoma boliviana	16,719	OM830311.1
Triatoma infestans	17,301	NC_035547.1
Triatoma dimidiata	17,019	NC_002609.1
Triatoma infestans	17,303	MZ350959.1
Triatoma rubrofasciata	17,15	MH934953.1
Triatoma infestans	17,301	KY640305.1
Triatoma huehuetenanguensis	15,601	OR611932.1
Triatoma huehuetenanguensis	15,603	OR611931.1
Triatoma dimidiata	15,444	OR611930.1
Triatoma sanguisuga	15,421	NC_050329.1
Triatoma huehuetenanguensis	15,91	NC_050325.1
Triatoma mazzottii	15,962	NC_050327.1
Triatoma mexicana	15,699	NC_050324.1
Triatoma migrans	17,323	NC_042881.1
Triatoma dimidiata	16,571	MT733872.1
Triatoma dimidiata	17,037	MT733873.1
Triatoma dimidiata	16,087	MT757852.1
Triatoma dimidiata	17,019	MT757851.1
Triatoma dimidiata	17,017	MT757850.1
Triatoma dimidiata	16,398	MT757849.1
Triatoma dimidiata	17,018	MT757848.1
Triatoma dimidiata	16,077	MT556666.1
Triatoma vitticeps	15,351	MT556665.1
Triatoma rubida	16,346	MT556664.1
Triatoma recurva	16,125	MT556663.1
Triatoma picturata	15,38	MT556661.1
Triatoma phyllosoma	15,72	MT556660.1
Triatoma dimidiata	15,44	MT556657.1
Triatoma dimidiata	15,925	MT556656.1
Triatoma sanguisuga	15,421	MT556653.1
Triatoma mazzottii	15,962	MT556651.1
Triatoma huehuetenanguensis	15,91	MT556649.1
Triatoma mexicana	15,699	MT556647.1
Triatoma infestans	15,767	MT561168.1
Triatoma migrans	17,323	MK770624.1
Triatoma lecticularia	16,067	NC_050326.1
Hospesneotomae protracta	15,519	MT556662.1
Meccus pallidipennis	15,564	MT556659.1
Meccus longipennis	15,703	MT556658.1

Hospesneotomae barberi	16,434	MT556655.1
Paratriatoma lecticularia	16,067	MT556650.1
Panstrongylus rufotuberculatus	16,331	NC_042682.1
Rhodnius pictipes	15908	NC_043846.1
Rhodnius prolixus	15,790	NC_050328.1

Table S3. GenBank accession numbers of mitochondrial gene sequences from *Mepraia* species

Genus	***species***	**IDs**	**Gene**	**Accession**
Mepraia	*gajardoi*	mg10	ATP6	PV521367
Mepraia	*gajardoi*	mg8	ATP6	PV521368
Mepraia	*gajardoi*	mg7	ATP6	PV521369
Mepraia	*gajardoi*	mg9	ATP6	PV521370
Mepraia	*gajardoi*	mg11	ATP6	PV521371
Mepraia	*gajardoi*	mg12	ATP6	PV521372
Mepraia	*parapatrica*	mp13	ATP6	PV521373
Mepraia	*parapatrica*	mp14	ATP6	PV521374
Mepraia	*parapatrica*	mp15	ATP6	PV521375
Mepraia	*parapatrica*	mp16	ATP6	PV521376
Mepraia	*parapatrica*	mp18	ATP6	PV521377
Mepraia	*parapatrica*	mp17	ATP6	PV521378
Mepraia	*spinolai*	ms1	ATP6	PV521379
Mepraia	*spinolai*	ms2	ATP6	PV521380
Mepraia	*spinolai*	ms3	ATP6	PV521381
Mepraia	*spinolai*	ms5	ATP6	PV521382
Mepraia	*spinolai*	ms4	ATP6	PV521383
Mepraia	*spinolai*	ms6	ATP6	PV521384
Mepraia	*gajardoi*	mg10	ATP8	PV521385
Mepraia	*gajardoi*	mg8	ATP8	PV521386
Mepraia	*gajardoi*	mg7	ATP8	PV521387
Mepraia	*gajardoi*	mg9	ATP8	PV521388
Mepraia	*gajardoi*	mg11	ATP8	PV521389
Mepraia	*gajardoi*	mg12	ATP8	PV521390
Mepraia	*parapatrica*	mp13	ATP8	PV521391
Mepraia	*parapatrica*	mp14	ATP8	PV521392
Mepraia	*parapatrica*	mp15	ATP8	PV521393
Mepraia	*parapatrica*	mp16	ATP8	PV521394
Mepraia	*parapatrica*	mp18	ATP8	PV521395
Mepraia	*parapatrica*	mp17	ATP8	PV521396
Mepraia	*spinolai*	ms1	ATP8	PV521397
Mepraia	*spinolai*	ms2	ATP8	PV521398
Mepraia	*spinolai*	ms3	ATP8	PV521399

Mepraia	*spinolai*	ms5	ATP8	PV521400
Mepraia	*spinolai*	ms4	ATP8	PV521401
Mepraia	*spinolai*	ms6	ATP8	PV521402
Mepraia	*gajardoi*	mg10	COI	PV521403
Mepraia	*gajardoi*	mg8	COI	PV521404
Mepraia	*gajardoi*	mg7	COI	PV521405
Mepraia	*gajardoi*	mg9	COI	PV521406
Mepraia	*gajardoi*	mg11	COI	PV521407
Mepraia	*gajardoi*	mg12	COI	PV521408
Mepraia	*parapatrica*	mp13	COI	PV521409
Mepraia	*parapatrica*	mp14	COI	PV521410
Mepraia	*parapatrica*	mp15	COI	PV521411
Mepraia	*parapatrica*	mp16	COI	PV521412
Mepraia	*parapatrica*	mp18	COI	PV521413
Mepraia	*parapatrica*	mp17	COI	PV521414
Mepraia	*spinolai*	ms1	COI	PV521415
Mepraia	*spinolai*	ms2	COI	PV521416
Mepraia	*spinolai*	ms3	COI	PV521417
Mepraia	*spinolai*	ms5	COI	PV521418
Mepraia	*spinolai*	ms4	COI	PV521419
Mepraia	*spinolai*	ms6	COI	PV521420
Mepraia	*gajardoi*	mg10	COII	PV521421
Mepraia	*gajardoi*	mg8	COII	PV521422
Mepraia	*gajardoi*	mg7	COII	PV521423
Mepraia	*gajardoi*	mg9	COII	PV521424
Mepraia	*gajardoi*	mg11	COII	PV521425
Mepraia	*gajardoi*	mg12	COII	PV521426
Mepraia	*parapatrica*	mp13	COII	PV521427
Mepraia	*parapatrica*	mp14	COII	PV521428
Mepraia	*parapatrica*	mp15	COII	PV521429
Mepraia	*parapatrica*	mp16	COII	PV521430
Mepraia	*parapatrica*	mp18	COII	PV521431
Mepraia	*parapatrica*	mp17	COII	PV521432
Mepraia	*spinolai*	ms1	COII	PV521433
Mepraia	*spinolai*	ms2	COII	PV521434
Mepraia	*spinolai*	ms3	COII	PV521435
Mepraia	*spinolai*	ms5	COII	PV521436
Mepraia	*spinolai*	ms4	COII	PV521437
Mepraia	*spinolai*	ms6	COII	PV521438
Mepraia	*gajardoi*	mg10	COIII	PV521439
Mepraia	*gajardoi*	mg8	COIII	PV521440
Mepraia	*gajardoi*	mg7	COIII	PV521441
Mepraia	*gajardoi*	mg9	COIII	PV521442

Mepraia	*gajardoi*	mg11	COIII	PV521443
Mepraia	*gajardoi*	mg12	COIII	PV521444
Mepraia	*parapatrica*	mp13	COIII	PV521445
Mepraia	*parapatrica*	mp14	COIII	PV521446
Mepraia	*parapatrica*	mp15	COIII	PV521447
Mepraia	*parapatrica*	mp16	COIII	PV521448
Mepraia	*parapatrica*	mp18	COIII	PV521449
Mepraia	*parapatrica*	mp17	COIII	PV521450
Mepraia	*spinolai*	ms1	COIII	PV521451
Mepraia	*spinolai*	ms2	COIII	PV521452
Mepraia	*spinolai*	ms3	COIII	PV521453
Mepraia	*spinolai*	ms5	COIII	PV521454
Mepraia	*spinolai*	ms4	COIII	PV521455
Mepraia	*spinolai*	ms6	COIII	PV521456
Mepraia	*gajardoi*	mg10	CytB	PV521457
Mepraia	*gajardoi*	mg8	CytB	PV521458
Mepraia	*gajardoi*	mg7	CytB	PV521459
Mepraia	*gajardoi*	mg9	CytB	PV521460
Mepraia	*gajardoi*	mg11	CytB	PV521461
Mepraia	*gajardoi*	mg12	CytB	PV521462
Mepraia	*parapatrica*	mp13	CytB	PV521463
Mepraia	*parapatrica*	mp14	CytB	PV521464
Mepraia	*parapatrica*	mp15	CytB	PV521465
Mepraia	*parapatrica*	mp16	CytB	PV521466
Mepraia	*parapatrica*	mp18	CytB	PV521467
Mepraia	*parapatrica*	mp17	CytB	PV521468
Mepraia	*spinolai*	ms1	CytB	PV521469
Mepraia	*spinolai*	ms2	CytB	PV521470
Mepraia	*spinolai*	ms3	CytB	PV521471
Mepraia	*spinolai*	ms5	CytB	PV521472
Mepraia	*spinolai*	ms4	CytB	PV521473
Mepraia	*spinolai*	ms6	CytB	PV521474
Mepraia	*gajardoi*	mg10	Nad1	PV521475
Mepraia	*gajardoi*	mg8	Nad1	PV521476
Mepraia	*gajardoi*	mg7	Nad1	PV521477
Mepraia	*gajardoi*	mg9	Nad1	PV521478
Mepraia	*gajardoi*	mg11	Nad1	PV521479
Mepraia	*gajardoi*	mg12	Nad1	PV521480
Mepraia	*parapatrica*	mp13	Nad1	PV521481
Mepraia	*parapatrica*	mp14	Nad1	PV521482
Mepraia	*parapatrica*	mp15	Nad1	PV521483
Mepraia	*parapatrica*	mp16	Nad1	PV521484
Mepraia	*parapatrica*	mp18	Nad1	PV521485

Mepraia	*parapatrica*	mp17	Nad1	PV521486
Mepraia	*spinolai*	ms1	Nad1	PV521487
Mepraia	*spinolai*	ms2	Nad1	PV521488
Mepraia	*spinolai*	ms3	Nad1	PV521489
Mepraia	*spinolai*	ms5	Nad1	PV521490
Mepraia	*spinolai*	ms4	Nad1	PV521491
Mepraia	*spinolai*	ms6	Nad1	PV521492
Mepraia	*gajardoi*	mg10	Nad2	PV521565
Mepraia	*gajardoi*	mg8	Nad2	PV521566
Mepraia	*gajardoi*	mg7	Nad2	PV521567
Mepraia	*gajardoi*	mg9	Nad2	PV521568
Mepraia	*gajardoi*	mg11	Nad2	PV521569
Mepraia	*gajardoi*	mg12	Nad2	PV521570
Mepraia	*parapatrica*	mp13	Nad2	PV521571
Mepraia	*parapatrica*	mp14	Nad2	PV521572
Mepraia	*parapatrica*	mp15	Nad2	PV521573
Mepraia	*parapatrica*	mp16	Nad2	PV521574
Mepraia	*parapatrica*	mp18	Nad2	PV521575
Mepraia	*parapatrica*	mp17	Nad2	PV521576
Mepraia	*spinolai*	ms1	Nad2	PV521577
Mepraia	*spinolai*	ms2	Nad2	PV521578
Mepraia	*spinolai*	ms3	Nad2	PV521579
Mepraia	*spinolai*	ms5	Nad2	PV521580
Mepraia	*spinolai*	ms4	Nad2	PV521581
Mepraia	*spinolai*	ms6	Nad2	PV521582
Mepraia	*gajardoi*	mg10	Nad3	PV521583
Mepraia	*gajardoi*	mg8	Nad3	PV521584
Mepraia	*gajardoi*	mg7	Nad3	PV521585
Mepraia	*gajardoi*	mg9	Nad3	PV521586
Mepraia	*gajardoi*	mg11	Nad3	PV521587
Mepraia	*gajardoi*	mg12	Nad3	PV521588
Mepraia	*parapatrica*	mp13	Nad3	PV521589
Mepraia	*parapatrica*	mp14	Nad3	PV521590
Mepraia	*parapatrica*	mp15	Nad3	PV521591
Mepraia	*parapatrica*	mp16	Nad3	PV521592
Mepraia	*parapatrica*	mp18	Nad3	PV521593
Mepraia	*parapatrica*	mp17	Nad3	PV521594
Mepraia	*spinolai*	ms1	Nad3	PV521595
Mepraia	*spinolai*	ms2	Nad3	PV521596
Mepraia	*spinolai*	ms3	Nad3	PV521597
Mepraia	*spinolai*	ms5	Nad3	PV521598
Mepraia	*spinolai*	ms4	Nad3	PV521599
Mepraia	*spinolai*	ms6	Nad3	PV521600

Mepraia	*gajardoi*	mg10	Nad4	PV521493
Mepraia	*gajardoi*	mg8	Nad4	PV521494
Mepraia	*gajardoi*	mg7	Nad4	PV521495
Mepraia	*gajardoi*	mg9	Nad4	PV521496
Mepraia	*gajardoi*	mg11	Nad4	PV521497
Mepraia	*gajardoi*	mg12	Nad4	PV521498
Mepraia	*parapatrica*	mp13	Nad4	PV521499
Mepraia	*parapatrica*	mp14	Nad4	PV521500
Mepraia	*parapatrica*	mp15	Nad4	PV521501
Mepraia	*parapatrica*	mp16	Nad4	PV521502
Mepraia	*parapatrica*	mp18	Nad4	PV521503
Mepraia	*parapatrica*	mp17	Nad4	PV521504
Mepraia	*spinolai*	ms1	Nad4	PV521505
Mepraia	*spinolai*	ms2	Nad4	PV521506
Mepraia	*spinolai*	ms3	Nad4	PV521507
Mepraia	*spinolai*	ms5	Nad4	PV521508
Mepraia	*spinolai*	ms4	Nad4	PV521509
Mepraia	*spinolai*	ms6	Nad4	PV521510
Mepraia	*gajardoi*	mg10	Nad4L	PV521511
Mepraia	*gajardoi*	mg8	Nad4L	PV521512
Mepraia	*gajardoi*	mg7	Nad4L	PV521513
Mepraia	*gajardoi*	mg9	Nad4L	PV521514
Mepraia	*gajardoi*	mg11	Nad4L	PV521515
Mepraia	*gajardoi*	mg12	Nad4L	PV521516
Mepraia	*parapatrica*	mp13	Nad4L	PV521517
Mepraia	*parapatrica*	mp14	Nad4L	PV521518
Mepraia	*parapatrica*	mp15	Nad4L	PV521519
Mepraia	*parapatrica*	mp16	Nad4L	PV521520
Mepraia	*parapatrica*	mp18	Nad4L	PV521521
Mepraia	*parapatrica*	mp17	Nad4L	PV521522
Mepraia	*spinolai*	ms1	Nad4L	PV521523
Mepraia	*spinolai*	ms2	Nad4L	PV521524
Mepraia	*spinolai*	ms3	Nad4L	PV521525
Mepraia	*spinolai*	ms5	Nad4L	PV521526
Mepraia	*spinolai*	ms4	Nad4L	PV521527
Mepraia	*spinolai*	ms6	Nad4L	PV521528
Mepraia	*gajardoi*	mg10	Nad5	PV521529
Mepraia	*gajardoi*	mg8	Nad5	PV521530
Mepraia	*gajardoi*	mg7	Nad5	PV521531
Mepraia	*gajardoi*	mg9	Nad5	PV521532
Mepraia	*gajardoi*	mg11	Nad5	PV521533
Mepraia	*gajardoi*	mg12	Nad5	PV521534
Mepraia	*parapatrica*	mp13	Nad5	PV521535

Mepraia	*parapatrica*	mp14	Nad5	PV521536
Mepraia	*parapatrica*	mp15	Nad5	PV521537
Mepraia	*parapatrica*	mp16	Nad5	PV521538
Mepraia	*parapatrica*	mp18	Nad5	PV521539
Mepraia	*parapatrica*	mp17	Nad5	PV521540
Mepraia	*spinolai*	ms1	Nad5	PV521541
Mepraia	*spinolai*	ms2	Nad5	PV521542
Mepraia	*spinolai*	ms3	Nad5	PV521543
Mepraia	*spinolai*	ms5	Nad5	PV521544
Mepraia	*spinolai*	ms4	Nad5	PV521545
Mepraia	*spinolai*	ms6	Nad5	PV521546
Mepraia	*gajardoi*	mg10	Nad6	PV521547
Mepraia	*gajardoi*	mg8	Nad6	PV521548
Mepraia	*gajardoi*	mg7	Nad6	PV521549
Mepraia	*gajardoi*	mg9	Nad6	PV521550
Mepraia	*gajardoi*	mg11	Nad6	PV521551
Mepraia	*gajardoi*	mg12	Nad6	PV521552
Mepraia	*parapatrica*	mp13	Nad6	PV521553
Mepraia	*parapatrica*	mp14	Nad6	PV521554
Mepraia	*parapatrica*	mp15	Nad6	PV521555
Mepraia	*parapatrica*	mp16	Nad6	PV521556
Mepraia	*parapatrica*	mp18	Nad6	PV521557
Mepraia	*parapatrica*	mp17	Nad6	PV521558
Mepraia	*spinolai*	ms1	Nad6	PV521559
Mepraia	*spinolai*	ms2	Nad6	PV521560
Mepraia	*spinolai*	ms3	Nad6	PV521561
Mepraia	*spinolai*	ms5	Nad6	PV521562
Mepraia	*spinolai*	ms4	Nad6	PV521563
Mepraia	*spinolai*	ms6	Nad6	PV521564

Table S4. Alignment statistics and best-fit substitution models for mitochondrial protein coding genes.

PCG	Length (bp)	Distinct Patterns	Parsimony - Informative Sites	Singleton Sites	Constant Sites	Best-Fit Model (AIC)
ATP6	651	49	93	0	558	TN+F+I
ATP8	147	40	79	3	65	TPM3+F+I
COI	1485	68	169	1	1315	TVM+F+I
COII	615	54	84	2	529	HKY+F+I
COIII	699	46	95	0	604	TIM3+F+I
CytB	1095	57	145	2	948	TN+F+I
Nad1	825	67	89	0	736	TN+F+R2
Nad2	912	58	102	1	809	TPM2+F+G4
Nad3	264	30	39	0	225	HKY+F+G4
Nad4	1284	102	159	4	1121	TN+F+G4
Nad4L	1284	102	159	4	1121	TN+F+G4
Nad5	1506	89	177	2	1327	TN+F+G4
Nad6	465	42	36	0	429	TPM3+F+I
