# Supplementary material for: Mitochondrial genomes from RNA-Seq reveal phylogeny and selection in Mepraia (Hemiptera: Reduviidae)
Source: Mol Genet Genomics. 2026 May 19;301(1):117. doi: 10.1007/s00438-026-02434-y (PMC13186837; doi:10.1007/s00438-026-02434-y)
Supplement: Supplementary file 4 — Supplementary file4 (PDF 445 KB) [file 438_2026_2434_MOESM4_ESM.pdf]

Recovery of Mitochondrial Genomes by RNA-Seq in *Mepraia*: Phylogeny, Natural Selection and Evolutionary Implications

Matheus Cardoso de Siqueira e Silva1, José Paulo Leite Guadanucci2; Tiago Belintani2
1Universidade de Araraquara (Uniara), Araraquara, SP, Brazil
2 São Paulo State University (Unesp), Rio Claro Arachnology Laboratory, Department of Biodiversity, Institute of Biosciences, Rio Claro, Brazil
Correspondence author:
Tiago Belintani,

tiagobellintani@gmail.com

Supplementary Figure S1. Phylogenetic trees inferred using maximum likelihood (ML) and Bayesian inference (BI) from mitochondrial protein-coding genes.

(A) Maximum likelihood tree based on the focal dataset.

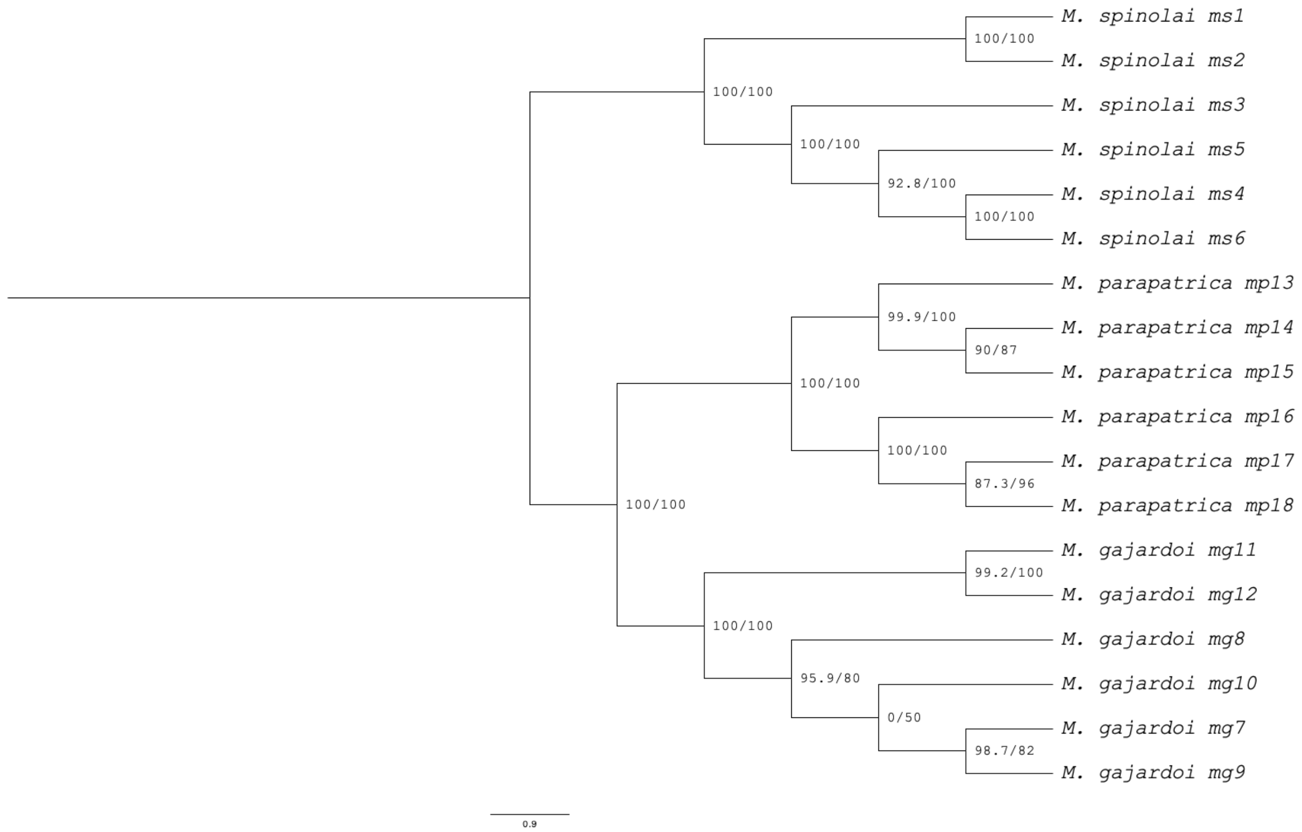

(B) Bayesian inference tree based on the focal dataset.

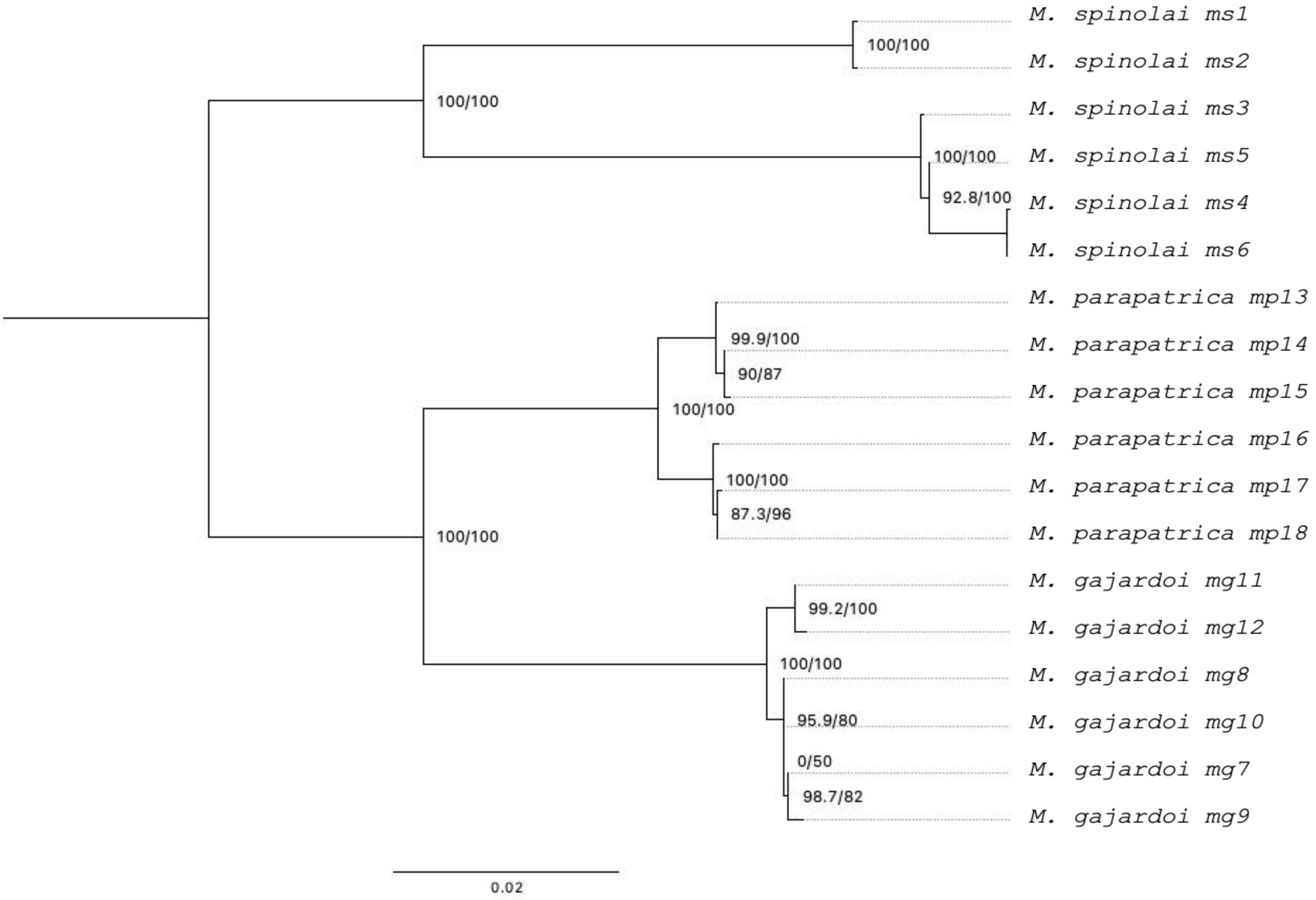

(C) Maximum likelihood tree based on the comprehensive dataset.

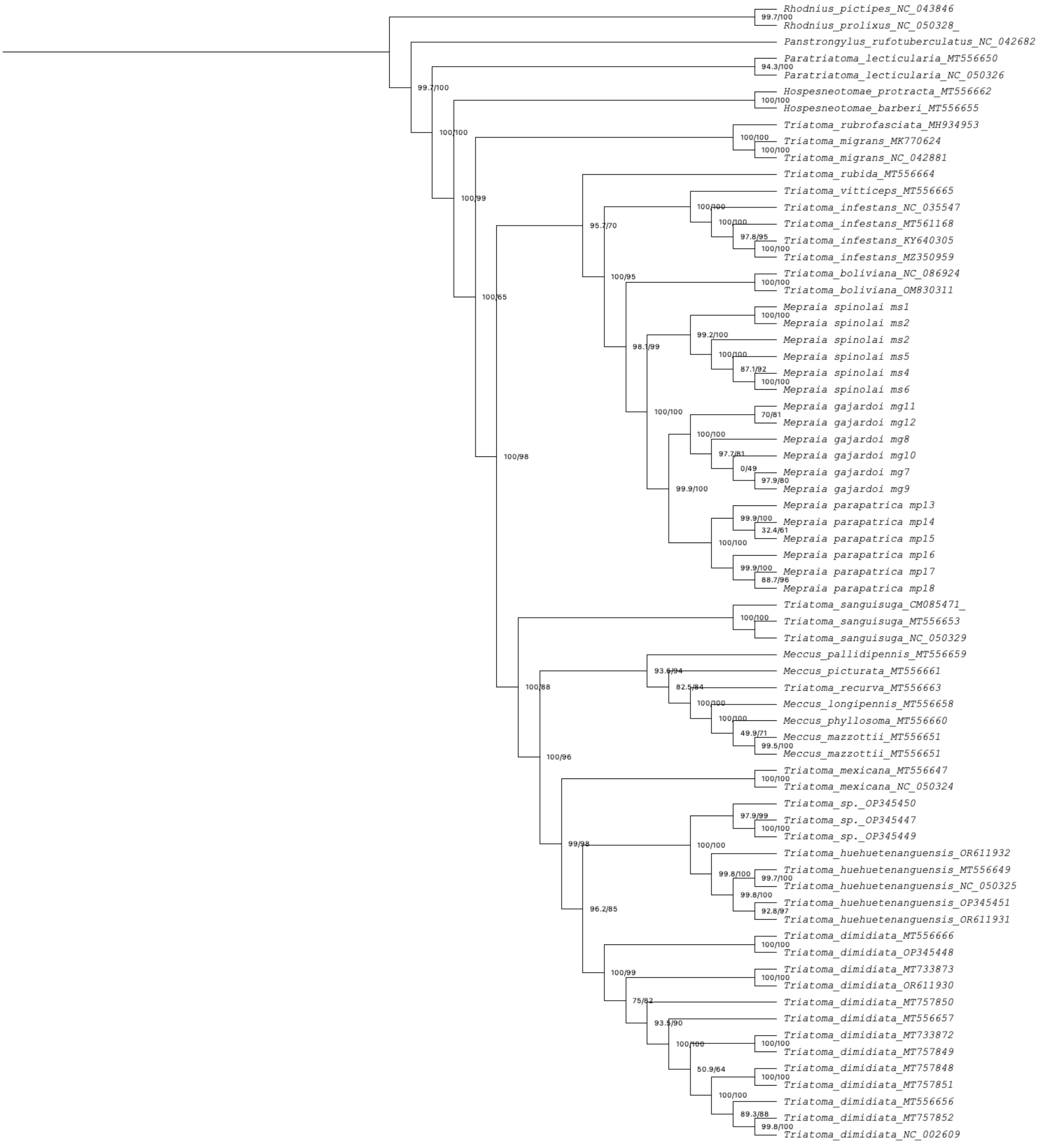

(D) Bayesian inference tree based on the comprehensive dataset.

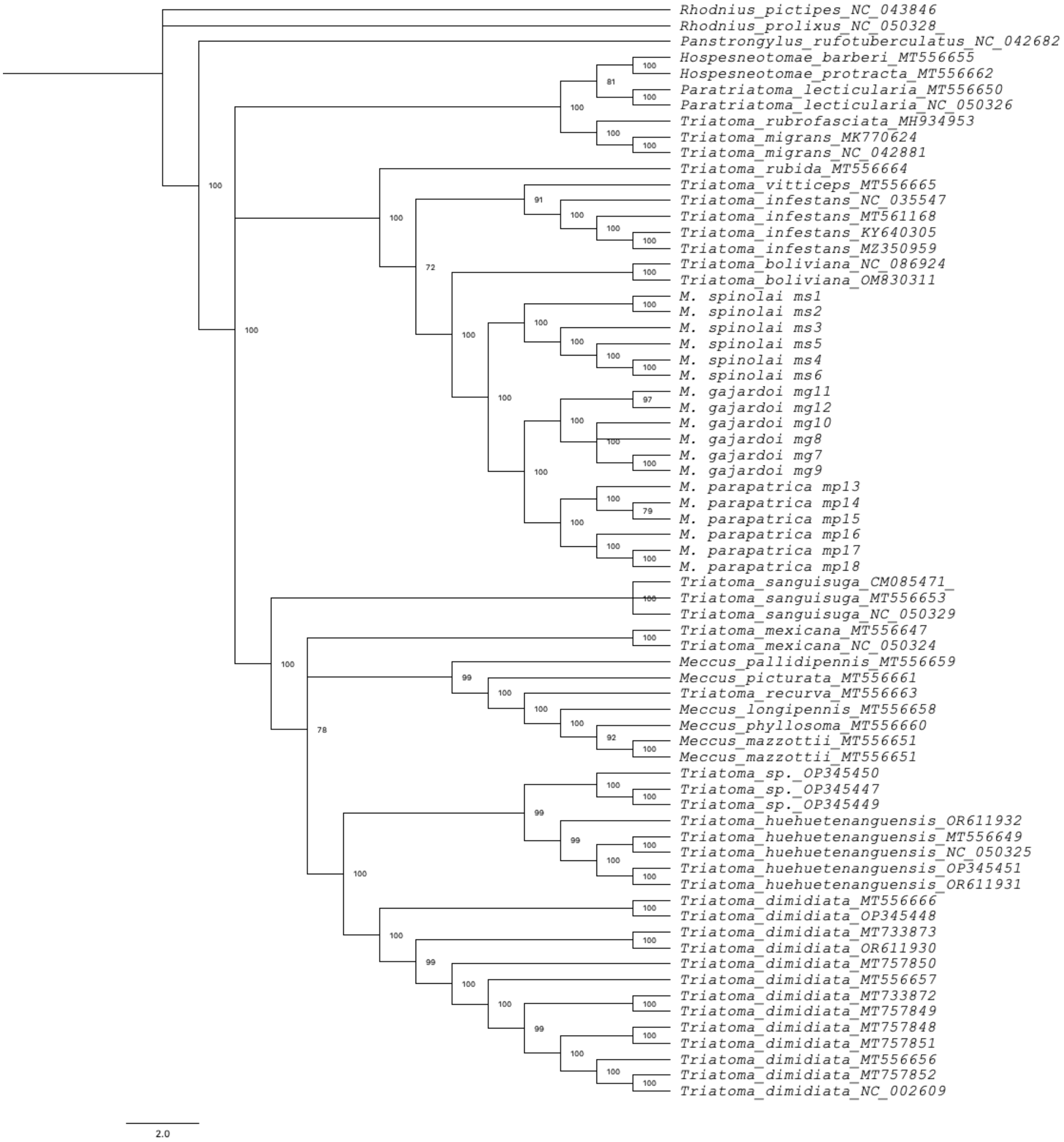
Rhodnius_pictipes_NC_043846
Rhodnius_prolixus_NC_050328_
Panstrongylus_rufotuberculatus_NC_042682
Hospesneotomae_barberi_MT556655
Hospesneotomae_protracta_MT556662
Paratriatoma_lecticularia_MT556650
Paratriatoma_lecticularia_NC_050326
Triatoma_rubrofasciata_MH934953
Triatoma_migrans_MK770624
Triatoma_migrans_NC_042881
Triatoma_rubida_MT556664
Triatoma_vitticeps_MT556665
Triatoma_infestans_NC_035547
Triatoma_infestans_MT561168
Triatoma_infestans_KY640305
Triatoma_infestans_MZ350959
Triatoma_boliviana_NC_086924
Triatoma_boliviana_OM830311
M. spinolai ms1
M. spinolai ms2
M. spinolai ms3
M. spinolai ms5
M. spinolai ms4
M. spinolai ms6
M. gajardoi mg11
M. gajardoi mg12
M. gajardoi mg10
M. gajardoi mg8
M. gajardoi mg7
M. gajardoi mg9
M. parapatrica mp13
M. parapatrica mp14
M. parapatrica mp15
M. parapatrica mp16
M. parapatrica mp17
M. parapatrica mp18
Triatoma_sanguisuga_CM085471_
Triatoma_sanguisuga_MT556653
Triatoma_sanguisuga_NC_050329
Triatoma_mexicana_MT556647
Triatoma_mexicana_NC_050324
Meccus_pallidipennis_MT556659
Meccus_picturata_MT556661
Triatoma_recurva_MT556663
Meccus_longipennis_MT556658
Meccus_phyllosoma_MT556660
Meccus_mazzottii_MT556651
Meccus_mazzottii_MT556651
Triatoma_sp._OP345450
Triatoma_sp._OP345447
Triatoma_sp._OP345449
Triatoma_huehuetenanguensis_OR611932
Triatoma_huehuetenanguensis_MT556649
Triatoma_huehuetenanguensis_NC_050325
Triatoma_huehuetenanguensis_OP345451
Triatoma_huehuetenanguensis_OR611931
Triatoma_dimidiata_MT556666
Triatoma_dimidiata_OP345448
Triatoma_dimidiata_MT733873
Triatoma_dimidiata_OR611930
Triatoma_dimidiata_MT757850
Triatoma_dimidiata_MT556657
Triatoma_dimidiata_MT733872
Triatoma_dimidiata_MT757849
Triatoma_dimidiata_MT757848
Triatoma_dimidiata_MT757851
Triatoma_dimidiata_MT556656
Triatoma_dimidiata_MT757852
Triatoma_dimidiata_NC_002609
2.0
